# Targeted intra-tumoral hyperthermia using uniquely biocompatible gold nanorods induces strong immunogenic cell death in two immunogenically ‘cold’ tumor models

**DOI:** 10.3389/fimmu.2024.1512543

**Published:** 2025-01-13

**Authors:** Barry E. Kennedy, Erin B. Noftall, Cheryl Dean, Alexander Roth, Kate N. Clark, Darren Rowles, Kulbir Singh, Len Pagliaro, Carman A. Giacomantonio

**Affiliations:** ^1^ Department of Pathology, Faculty of Medicine, Dalhousie University, Halifax, NS, Canada; ^2^ Department of Diagnoses, Sona Nanotech Inc.™, Halifax, NS, Canada; ^3^ Department of R&D, Sona Nanotech Inc.™, Halifax, NS, Canada; ^4^ Department of Surgery, Faculty of Medicine, Dalhousie University, Halifax, NS, Canada

**Keywords:** gold nanorods, hyperthermia, immunotherapy, interleukin-2, photothermal therapy, breast cancer, melanoma

## Abstract

**Introduction:**

Hyperthermia is an established adjunct in multimodal cancer treatments, with mechanisms including cell death, immune modulation, and vascular changes. Traditional hyperthermia applications are resource-intensive and often associated with patient morbidity, limiting their clinical accessibility. Gold nanorods (GNRs) offer a precise, minimally invasive alternative by leveraging near-infrared (NIR) light to deliver targeted hyperthermia therapy (THT). THT induces controlled tumor heating, promoting immunogenic cell death (ICD) and modulating the tumor microenvironment (TME) to enhance immune engagement. This study explores the synergistic potential of GNR-mediated THT with immunotherapies in immunogenically ‘cold’ tumors to achieve durable anti-tumor immunity.

**Methods:**

GNRs from Sona Nanotech Inc.™ were intratumorally injected and activated using NIR light to induce mild hyperthermia (42–48°C) for 5 minutes. Tumor responses were analyzed for cell death pathways and immune modulation. The immunogenic effects of THT were assessed alone and in combination with intratumoral interleukin-2 (i.t. IL-2) or systemic PD-1 immune checkpoint blockade. Immune cell infiltration, gene expression changes, and tumor growth kinetics were evaluated.

**Results:**

THT reduced tumor burden through cell death mechanisms, including upregulated ICD marked by calreticulin exposure within 48 hours. By 48 hours, CD45+ immune cell levels were increased, including increased levels of immunosuppressive M2 macrophages. While THT led to innate immune cell stimulations highlighted by gene expression upregulation in the STING cGAS pathway and enhanced M1 and dendritic cell levels, tumor regrowth was observed within six days post-treatment. To enhance THT's immunogenic effects, the therapy was combined with intratumoral interleukin-2 (i.t. IL-2) or systemic PD-1 immune checkpoint blockade. Sequential administration of i.t. IL-2 post-THT induced robust CD8+ T-cell infiltration and led to sustained tumor regression in both treated and distant tumors, accompanied by the emergence of memory T cells. However, IL-2-induced immunosuppressive T-reg populations were also sustained to tumor endpoint suggesting that therapy could be further enhanced. Additionally, PD-1 expression, which was upregulated in CD8+ T cells by THT, was targeted with systemic PD-1 inhibition, further augmenting immune engagement within the TME.

**Discussion:**

These combinatory treatments demonstrated synergistic effects, promoting durable anti-tumor responses and immune memory. Collectively, GNR-mediated THT effectively reduces tumor burden and remodels the TME, potentiating systemic immunity and enhancing the impact of complementary immunotherapies.

## Graphical Abstract

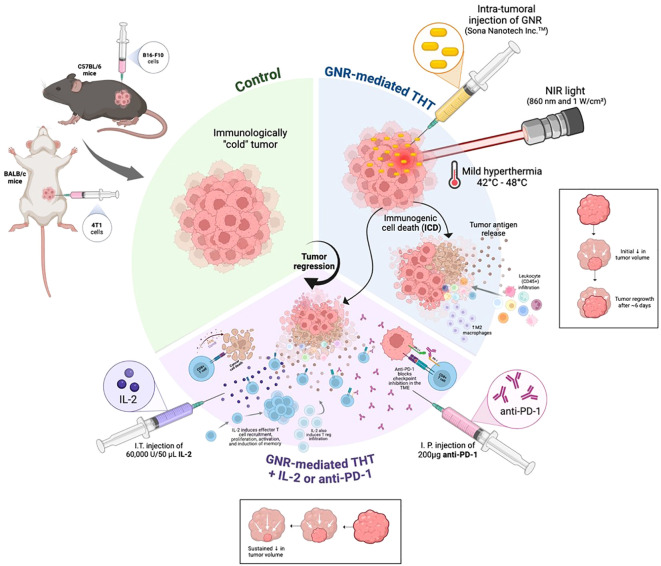

## Introduction

Hyperthermia as an adjunct in the multimodality treatment (surgery, chemotherapy, and radiotherapy) for several cancers has been well established, with varying degrees of clinical efficacy and long-term benefit reported depending on the specific cancer being treated (1). Isolated hyperthermic limb perfusion and limb infusions, used for the treatment of locoregionally advanced melanoma and sarcoma, and hyperthermic isolated peritoneal chemoperfusion for advanced peritoneal based cancers are two examples of hyperthermia-based cancer treatments still widely used in clinical practice today (2, 3). Several mechanisms have been proposed to explain the clinical responses to hyperthermic stress in cancer treatment, including cell death pathways, immune modulation, and vascular changes (4–6). However, there is no consensus regarding a single dominant mechanism responsible for these effects, as multiple factors likely contribute. This complexity underscores the need for further research to clarify the precise contributions of each mechanism.

Currently, protocols for the application of hyperthermia in cancer treatment are highly complex and resource intensive. Consequently, applications of hyperthermia are generally limited to single treatment exposures, and generally associated with significant additional cost, additional patient morbidity, and potentially patient mortality (7). Accordingly, the application of hyperthermia in cancer treatment is limited to major centers with adequate resources and infrastructure in place to support the additional costs, and with patient supports in place necessary to deliver this type of treatment safely and effectively. As a result, many patients are unable to access the potential benefits of hyperthermia in the treatment of their cancer. There is an urgent need for more elegant, less toxic, and less resource intensive methods of delivering hyperthermia in the clinical setting. Moreover, a more precise understanding of the mechanism of action for hyperthermia as an effective adjunct to current cancer treatment regimens is critical.

To address these challenges, we utilize the precise application of targeted hyperthermia therapy (THT) to tumor microenvironments (TME), leveraging near-infrared (NIR) light to induce localized, gentle heating of tumors (8). Unlike traditional hyperthermia, which is typically ablative thus potentially associated with widespread tissue damage, THT provides a highly controlled rise in tumor temperature (targeting between 42°C and 48°C) that selectively induces apoptotic cell death in tumor cells while minimizing harm to surrounding healthy tissues (9). The molecular mechanisms (in part) underlying this process involve the activation of heat shock proteins, disruption of cellular homeostasis, and induction of oxidative stress, leading to irreversible damage to cancer cells (10). Additionally, THT modulates the TME, enhances tumor perfusion, oxygenation, and immune cell infiltration, which collectively improves the delivery and efficacy of concurrent therapies (11).

Gold nanoparticles (GNPs), especially gold nanorods (GNRs), have emerged as highly effective agents for THT due to their unique optical properties, which enable efficient conversion of NIR light into heat (12). GNRs can be engineered to absorb light at specific wavelengths, making them ideal for deep tissue penetration and targeted tumor destruction. For example, GNRs have been reported to cause hyperthermic-dependent tumor death in several cancers (13). Li et al. developed a targeted GNR system combining photothermal therapy, photodynamic therapy, and chemotherapy, which effectively inhibited breast cancer tumor growth and metastasis through synergistic mechanisms (14). Ali et al. demonstrated that GNR-assisted photothermal therapy induced cytochrome c and p53 activation, leading to tumor apoptosis while avoiding necrosis in a preclinical model of squamous cell carcinoma (15). Zhang et al. showed that GNR-mediated photothermal therapy induces temperature-dependent melanoma cell death, apoptosis at mild hyperthermic temperatures, and necrosis at temperatures above 49°C via the RIPK1 pathway (16). Traditionally, GNRs are supplied in the form of aqueous dispersion and manufacturing requires the use of a cytotoxic surfactant cetyltrimethylammonium bromide (CTAB). CTAB serves as a shape directing and dispersion stabilizing agent. Ligand exchange of CTAB with other molecules is only partially successful in removing CTAB, and the residual CTAB remains a concern for *in vivo* use, particularly long-term. Sona Nanotech Inc.™ offers a superior solution by completely eliminating CTAB from their GNR manufacturing process without any compromise of stability or performance efficacy of GNRs.

Research has demonstrated that hyperthermia can reduce tumor burden and initiate an immunogenic cell death (ICD) response, and these effects can be further amplified when combined with systemic immunotherapy (17). Additionally, novel immunotherapeutic approaches such as intra-tumoral (i.t.) injections of interleukin-2 (IL-2, referred to as “i.t. IL-2”), have demonstrated efficacy in enhancing the body's immune response against cancers (18–22). The potential synergy between THT and immunotherapy lies in their complementary mechanisms: THT induces tumor cell death, thereby exposing novel tumor-specific antigens to the innate immune system. Depending on the robustness of the tumor neo-antigens the resultant immunity could theoretically be sufficient to clear all cells bearing the same antigenic signals. Furthermore, THT has been shown to alter the immunosuppressive TME, facilitating a more effective immune response and overcoming resistance to immunotherapy (23). However, although both THT and i.t. immunotherapies like IL-2 have individually shown promise in cancer treatment, the mechanism of their synergistic potential remain largely unexplored. This gap in research highlights the need to better understand how these therapies can work together to enhance anti-tumor immune efficacy and achieve more comprehensive therapeutic outcomes.

In this study, we demonstrate in two distinctly different, immunogenically ‘cold’ preclinical models (4T1 and B16-F10) that by leveraging the unique properties of GNRs to transform NIR light into thermal energy for the precise delivery of THT into TMEs, we successfully activate ICD and tumor-directed innate immunity. The choice of using two distinct murine species, such as 4T1 (breast cancer) and B16-F10 (melanoma), was guided by the need to evaluate the therapeutic strategy across different TMEs, growth kinetics, and immune profiles. Furthermore, we show that the subsequent addition of immunotherapies (i.t., and / or systemic immunotherapy) in the context of this novel THT-induced, tumor-specific immunity results in synergistic and highly desired abscopal immune responses.

## Materials and methods

### Animals

6-8 week old Female BALB/c mice and female C57BL/6 mice were purchased from Charles River Laboratories (Montreal, Canada) and acclimated for one week at the Carleton Animal Care Facility at Dalhousie University, Halifax, NS, Canada. Mice were housed in ventilated rack cages under a standard 12-hour light/dark cycle, with a controlled room temperature of 22°C and humidity of 55–60%. Animals were fed a standard diet of rodent chow and water *ad libitum*. Animal weight was recorded throughout the study. This study was conducted in accordance with the guidelines and standards set forth by the Canadian Council on Animal Care and approved by the University Committee on Laboratory Animals at Dalhousie University (#23-081). Before tumor cell implantation, fur was shaved in the areas where tumor cells were to be implanted. A total of 94 4T1 tumor-bearing BALB/c mice were used across this study, allocated as follows: 29 control, 33 THT, 14 i.t. IL-2, 12 THT + i.t. IL-2, 2 PD-1, and 4 PD-1 + THT. Additionally, 40 B16-F10 tumor-bearing C57BL/6 mice were included, divided into 11 control, 12 THT, 7 i.t. IL-2, and 10 THT + i.t. IL-2 groups. These mice were distributed across four independent experiments for the 4T1 model and two independent experiments for the B16-F10 model. Each study had different endpoint goals, such as varying time on study and distinct downstream analysis objectives. Exact numbers of mice used in each type of analysis are provided in the respective figure legends.

### Cell culture

4T1 and B16-F10 cells (ATCC) were cultured under standard conditions in a humidified incubator at 37°C with 5% CO_2_. 4T1 cells, derived from the mammary gland tissue of a BALB/c strain mouse, are a widely used breast cancer cell line that serves as a model for metastatic and triple-negative breast cancer (24). B16-F10 cells, originally derived from C57BL/6 mouse melanoma, are commonly used to model melanoma and facilitate the study of tumor immunology and metastasis (25). 4T1 cells were maintained in RPMI-1640 medium (Gibco) supplemented with 10% fetal bovine serum (FBS, Gibco) and 1% penicillin-streptomycin (Gibco). B16-F10 cells were cultured in Dulbecco's Modified Eagle Medium (DMEM, Gibco) supplemented with 10% FBS and 1% penicillin-streptomycin.

### Tumor establishment

6-8 week old BALB/c mice, were subcutaneously (s.c.) injected into the mammary pad on the left side with 1 × 10^5^ 4T1 cells in 100 µL of phosphate buffered saline (PBS, Gibco), while 5 × 10^5^ B16-F10 melanoma cells in 100 µL of PBS were injected s.c. into the left flank of 6-8 week old C57BL/6 mice, all under isoflurane anesthesia. To evaluate a systemic response to the treatments, 5 × 10^4^ 4T1 cells were injected into the right fourth mammary pad of BALB/c mice to establish a contralateral tumor that would not be directly targeted by i.t. therapies or exposed to NIR light. The 72-hour interval before injecting 4T1 cells into the right fourth mammary pad of BALB/c mice was implemented to allow the tumor to develop into a suitable size for targeted treatment while acting as a delayed model compared to the primary tumor. This procedure was performed only in the study designed to measure the systemic effects of the treatments. Tumor volume was measured using a digital caliper and calculated as an ellipsoid (length x width x height x 1/2).

### Biochemical parameters, synthesis, and toxicity assessment of Sona Nanotech Inc.™ GNRs

#### Synthesis and functionalization

GNRs were synthesized using an in-house developed wet chemical synthesis method. Briefly, gold salt was dissolved in an aqueous solution containing a proprietary surfactant blend. The gold salt was then reduced to its zero-oxidation state, and proprietary conditions were applied to facilitate the growth of gold crystals into a rod-shaped morphology. Following synthesis, the majority of native surfactants in the GNR dispersion were replaced with low-molecular-weight polyethylene glycol (PEG) to enhance biocompatibility and stability.

#### Physicochemical characterization

The size and morphology of GNRs were analyzed using transmission electron microscopy (TEM). GNR samples were diluted 50-fold with ultrapure water, and 2.5 μL was applied to glow-discharged carbon-coated copper grids. After air drying, samples were imaged using a T-12 TEM (FEI) operating at an acceleration voltage of 120 keV. Hydrodynamic size measurements were performed using a Malvern Zetasizer Nano ZS instrument equipped with a back-scattering detector (173°). Measurements were conducted at 25°C in ultrapure water, 10 mM NaCl (for zeta potential determination), and PBS (to mimic physiological ionic strength), following NIST-NCL protocol PCC-1 (26).

Reversed-phase high-performance liquid chromatography (RP-HPLC) with charged aerosol detection was used to quantify PEG and surfactant concentrations. Chromatography was conducted using a Zorbax 300SB-C18 column with acetonitrile and water containing trifluoroacetic acid (TFA) as the mobile phases. For PEG quantification, GNRs were dissolved with potassium cyanide (1 M), and calibration standards were prepared using 10 kDa PEG at concentrations ranging from 10 to 100 μg/mL. Surfactant concentrations were similarly quantified, using calibration standards ranging from 15 to 300 μg/mL for two proprietary surfactants.

#### Endotoxin testing

Endotoxin levels were measured using chromogenic and turbidity Limulus Amebocyte Lysate (LAL) assays, following Nanotechnology Characterization Laboratory (NCL) protocols (STE-1.1 and STE-1.2) (27). The endotoxin limit (EL) and maximum valid dilution (MVD) were calculated according to United States Pharmacopeia (USP) standards (BET85). The EL was calculated according to the formula: EL = K/M where K is the USP acceptable endotoxin limit and M is the maximum dose. The amount of endotoxin allowed per dose per hour (K) for all routes of administration, except intrathecal, is 5 EU/kg/hr. The maximum dose (M) was calculated using the efficacious dose of 13.2 mg/kg of Au. This maximum tested mouse dose was converted into a human equivalent dose (HED) using the formula shown below, to provide the maximum dose (M) of 1.07 mg/kg.


$$\begin{array}{c} \text{HED}=\text{Mouse Dose }÷12.3\text{ M }=13.2\text{ mg}/\text{kg }÷12.3\;\\ =1.07\text{ mg}/\text{kg} \end{array}$$

Therefore, EL = K/M EL = 5 EU/kg/hr ÷ 1.07 mg/kg = 4.66 EU/mg/hr.

### Study design

In this study, we used two distinct preclinical tumor models, 4T1 and B16-F10, to evaluate the therapeutic effects of GNR-mediated THT and immune modulation through IL-2 and anti-PD-1 therapies. Once tumors reached a palpable size (~50 mm³, approximately 10 days post-cell implantation), treatments were administered (see **Tables 1**, **2**). GNRs (100 µL, 200 µg GNR) or PBS (100 µL, Gibco) were injected directly into the tumor. Control (PBS) and GNR-injected tumors were subsequently exposed to NIR (860 nm and 1 W/cm²). IL-2 was administered at 3 doses i.t. at a concentration of 60,000 U/50 µL (Biolegend) (28), and anti-PD-1 (BioXcell) was administered at three does i.p. at a dose of 200µg per injection (29).

**Table 1:** Treatment schedule for 4T1 model.

Groups	Treatment	Day -1	Day 0	Day 1	Day 3	Day 5
1	Control	i.t. PBS	NIR	i.t. PBS	i.t. PBS	i.t. PBS
2	THT	i.t. GNR	NIR	i.t. PBS	i.t. PBS	i.t. PBS
3	IL-2	i.t. PBS	NIR	i.t. IL-2	i.t. IL-2	i.t. IL-2
4	Anti-PD-1	i.t. PBS	NIR	i.p. αPD-1	i.p. αPD-1	i.p. αPD-1
5	THT + IL-2	i.t. GNR	NIR	i.t. IL-2	i.t. IL-2	i.t. IL-2
6	THT + αPD-1	i.t. GNR	NIR	i.p. αPD-1	i.p. αPD-1	i.p. αPD-1

**Table 2:** Treatment schedule for B16-F10 model.

Groups	Treatment	Day -1	Day 0	Day 2	Day 4
1	Control	i.t. PBS	NIR	i.t. PBS	i.t. PBS
2	THT	i.t. GNR	NIR	i.t. PBS	i.t. PBS
3	IL-2	i.t. PBS	NIR + i.t. IL-2	i.t. IL-2	i.t. IL-2
4	THT + IL-2	i.t. GNR	NIR + i.t. IL-2	i.t. IL-2	i.t. IL-2

### NIR protocol

NIR treatment was administered using a LDX Laser (Model: LDX-3520-860-HHLFC, Minnetronix) 24 hours following the i.t. injection of either GNR or PBS. The laser was positioned approximately 2 cm above the tumor surface, delivering an intensity of 1 W/cm² (30, 31). The internal temperature of the GNR-treated tumors was maintained between 42°C and 48°C for five minutes. Temperature was closely monitored throughout and maintained with an on and off cycle. To monitor the therapeutic effect, external skin temperature was measured using a thermal camera (HIKmicro), and internal tumor temperature was tracked with intra-tumoral temperature probes (OMEGA) to ensure adequate thermal conditions were maintained. Both GNR-injected tumors and control tumors were exposed to the same NIR treatment protocol; however, only GNR-treated tumors reached the hyperthermic range. To prevent thermal damage, aloe vera gel was applied to the treated skin area before NIR exposure.

### Flow cytometry

Prior to tumor removal, mice were euthanized using CO_2_ overdose on anesthetized mice. After euthanasia, tumors were carefully excised using sterile surgical techniques. The tumors were then processed into single-cell suspensions using a mouse tumor dissociation kit and a gentleMACS™ Dissociator (Miltenyi Biotec) for subsequent flow cytometry analysis. Cells were prepared for flow cytometric analysis using lymphocyte and myeloid panels in Horizon^TM^ Brilliant Stain Buffer (BD) with the appropriate fluorochrome-conjugated antibodies, as detailed in **Table 3**. Stained cells were resuspended in FACS buffer (PBS containing 2% FBS) for analysis. Flow cytometry was performed on a FACSCelesta (BD Biosciences), and data was collected. The collected data were analyzed using FlowJo software (FlowJo LLC), with gating strategies applied to identify and quantify the relevant cell populations.

**Table 3:** Antibodies for flow cytometry.

Panel	Antigen	Fluorophore	Company
Lymphoid	CD3	APC	BD
Lymphoid	CD4	APC-Cy7	BD
Lymphoid	CD8	FITC	BD
Lymphoid	CD25	BV650	BD
Lymphoid	FOXP3	PE	BD
Lymphoid	CD27	BV421	BD
Lymphoid	CD19	AF700	BD
Lymphoid	CD44	BV510	BD
Lymphoid	CD62L	BV786	BD
Lymphoid	NK1.1	PE-Cy5.5	BD
Lymphoid	PD1	BV605	BD
Myeloid	F4/80	APC	BD
Myeloid	CD45	APC-Cy7	BD
Myeloid	CD273	FITC	BD
Myeloid	CD206	BV650	BD
Myeloid	PDL1	PE	BD
Myeloid	MHCII IA/IE	BV421	BD
Myeloid	CD11c	AF700	BD
Myeloid	CD86	BV510	BD
Myeloid	CD11b	BV786	BD
Myeloid	CD80	PE-Cy5.5	BD
Myeloid	Ly6G	BV605	BD
Myeloid	Ly6C	PECy7	BD
–	Calreticulin	AF647	Abcam
–	Fixable Viability Stain 510	BV510	BD

### Quantitative PCR

Tumors were harvested and immediately snap-frozen in liquid nitrogen for subsequent RNA extraction. RNA was isolated from the samples using the PureLink™ RNA Mini Kit (Invitrogen) following the manufacturer's protocol. The quantity and purity of the isolated RNA were assessed using a NanoDrop™ spectrophotometer (Thermo Fisher Scientific). Subsequently, RNA was reverse transcribed into complementary DNA (cDNA) using the iScript™ cDNA Synthesis Kit (Bio-Rad) according to the manufacturer's instructions.

For qPCR analysis, specific primers were designed and validated for genes of interest, as listed in **Table 4**. Reactions were set up using SYBR™ Green PCR Master Mix (Invitrogen) protocol. The relative expression levels were normalized to GAPDH and calculated using the 2^(-ΔΔCt) method (32).

**Table 4:** qPCR primer sequences.

Target	Forward (5’ to 3’)	Reverse (5’ to 3’)
Ccl7	CTGCTCTCCAGCGCTCTCA	GTAAGAAAAGCAGCAGGCGG
Cxcl10	AAGTGCTGCCGTCATTTTCT	GTGGCAATGATCTCAACACG
Cxcl9	TGTGGAGTTCGAGGAACCCT	TGCCTTGGCTGGTGCTG
IFNa1	CTACTGGCCAACCTGCTCTC	CCTTCTTGATCTGCTGGGCA
IFNa4	CCTGTGTGATGCAGGAACC	TCACCTCCCAGGCACAGA
IL12	ACCCTGACCATCACTGTCAA	GTGGAGCAGCAGATGTGAGT
IL18	CTGGCCGTGGCTCTCTTG	CCTTGGCAAAACTGCACCTT
IL1a	GAGAGCCGGGTGACAGTATC	TGACAAACTTCTGCCTGACG
IL1b	CTGCAGCTGGAGAGTGTGG	GGGGAACTCTGCAGACTCAA
IL6	AGTTGCCTTCTTGGGACTGA	TCCACGATTTCCCAGAGAAC
IRF3	GGCTTGTGATGGTCAAGGTT	CATGTCCTCCACCAAGTCCT
Isg15	TGTGAGAGCAAGCAGCCAGA	CCCCCAGCATCTTCACCTTT
NLRP3	AGAAGAGACCACGGCAGAAG	CCTTGGACCAGGTTCAGTGT
Oas1a	CTGCATCAGGAGGTGGAGTT	ACTCGGGAACCATCCTTTTT
Stat1	GGCCTCTCATTGTCACCGAA	TACCACAGGATAGACGCCCA
TLR7	AATCCACAGGCTCACCCATA	CAGGTACCAAGGGATGTCCT
TLR8	GACATGGCCCCTAATTTCCT	GACCCAGAAGTCCTCATGGA
TLR9	CCAGACGCTCTTCGAGAACC	GTTATAGAAGTGGCGGTTGT

### Immunohistochemistry

Flash-frozen tumors from 4T1 tumor-bearing mice treated with control, i.t. IL-2, THT alone, or THT+ i.t. IL-2 were processed and embedded in optimal cutting temperature (OCT) compound mixed with sucrose. Slides were fixed in ice-cold acetone. For immunohistochemical staining, slides were incubated with Anti-CD3 epsilon antibody [SP7] (Abcam) and counterstained with hematoxylin. All staining procedures were performed by Dalhousie University's Department of Pathology, Histology and Research Services Lab. Representative images taken at 40x.

### Statistical analysis

Statistical analyses were performed on the collected data to assess the significance of differences between treatment effects. For comparisons between two groups, an unpaired two-tailed Student’s t-test was used. When comparing three or more groups, one-way analysis of variance (ANOVA) was conducted, followed by Tukey's multiple comparison test to identify specific group differences. All statistical tests were performed using GraphPad Prism version 10.4.1 for Windows, GraphPad Software, Boston, Massachusetts USA, www.graphpad.com. A p-value of less than 0.05 was considered statistically significant. Data are presented as mean ± standard deviation (SD) unless otherwise specified.

## Results

### Biochemical parameters and toxicity assessment of Sona Nanotech Inc.^TM^ GNRs

**Figure 1:**
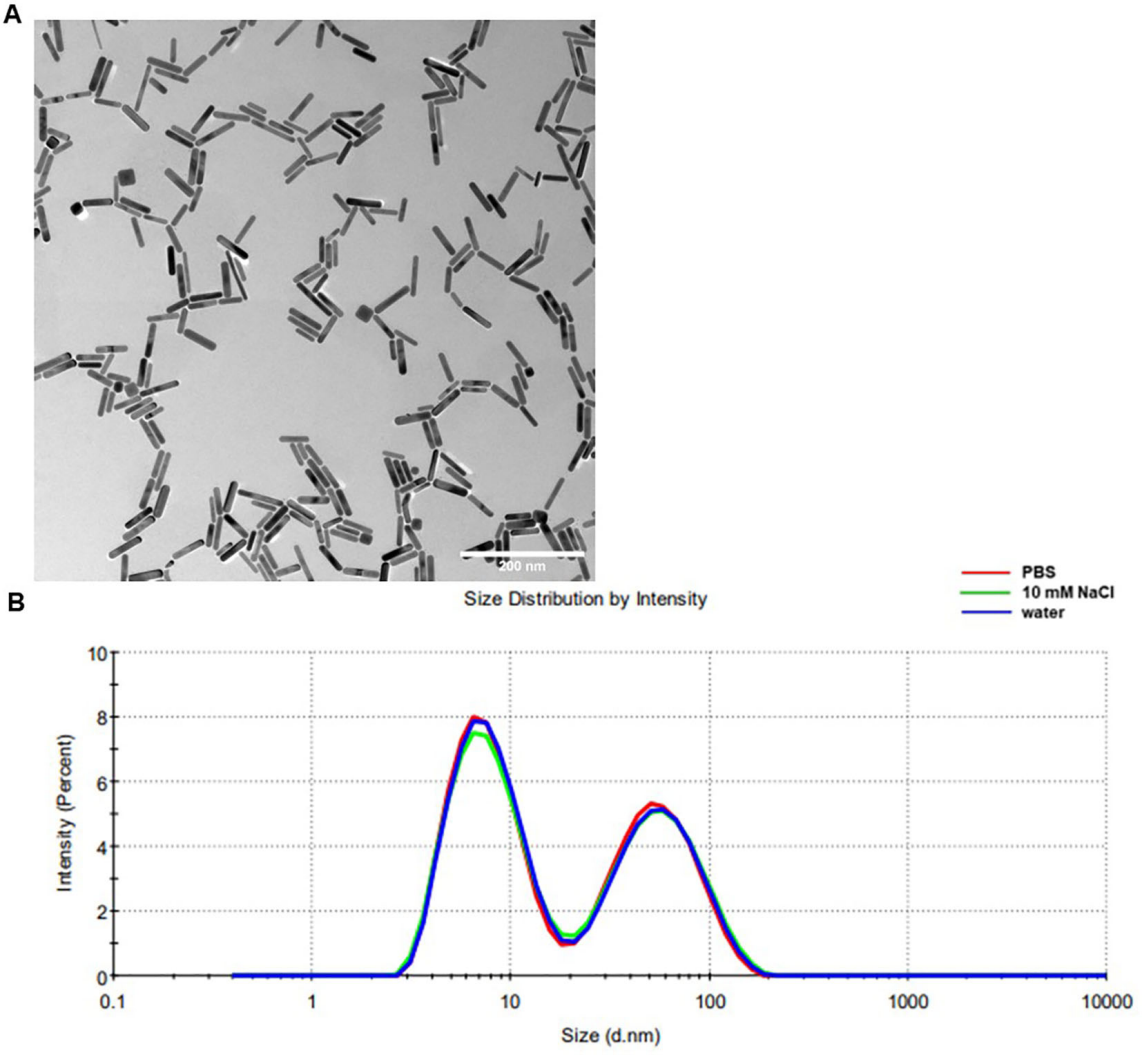
Biochemical properties of Sona Nanotech^TM^’s GNRs. Representative TEM images of GNRs samples **(A)**, measurement bar is 200 nm. Images were taken at 100,000× magnification. **(B)** Representative DLS for a GNR sample diluted in different media, showing size distribution by intensity.

TEM analysis (**Figure 1A**) demonstrated a high degree of particle uniformity, consistent with commercially available GNRs of comparable dimensions (**Table 5**) (33). The measured size distribution aligned with typical values for this nanoparticle type, underscoring the reproducibility of the synthesis process. DLS analysis (**Figure 1B**, **Table 6**) indicated slightly larger hydrodynamic particle sizes relative to TEM, attributable to contributions from surface coatings and solvation layers inherent to the DLS measurement technique (34).

**Table 5:** Particle analysis of TEM data of **Figure 1A**.

Shape	Particle count	Percentage	Primary Axis (nm)	Secondary Axis (nm)	Aspect Ratio
Sona Nanotech Inc.™					
Rod shape	319	79.90%	51.1 ± 10.8	12.2 ± 1.9	4.2± 0.9
Non-Rod shape		20.10%	22.4 ± 3.4	20.6 ± 3.1	1.09± 0.08
Commercial GNRs					
Rod shape	323	78.00%	50.0 ± 5.0	11 ± 2.0	4.5± 0.8
Non-Rod shape		22.00%	26.0 ± 3.0	21.0 ± 3.0	1.08± 0.07

**Table 6:** DLS results for GNR samples diluted in different media.

Dilution	Z-Avg (nm)	PDI	Int-Peak (nm)	% Int	Vol-Peak (nm)	%Vol
10-fold, PBS	12.0 ± 0.1	0.424 ± 000.2	58.5 ± 2.4	44.6 ± 1.2	5.4 ± 0.2	99.9 ± 0.1
10-fold, 10mM NaCl	12.3 ± 0.1	0.431 ± 000.4	61.2 ± 2.6	44.7 ± 1.5	5.4 ± 0.3	99.9 ± 0.2
10-fold, Water	12.2 ± 0.1	0.423 ± 000.3	61.0 ± 2.4	43.5 ± 1.5	5.4 ± 0.4	99.9 ± 0.3

XPS analysis confirmed uniform PEG functionalization across the GNR population, with PEG density quantified to ensure consistent surface coverage. RP2-HPLC was employed to assess the chemical composition of the GNR dispersion phase (**Table 7**), revealing that the concentrations of residual surfactants and reagents were significantly below reported LD50 values. CTAB, a cytotoxic surfactant, was included as a reference to simulate worst-case safety scenarios for evaluation purposes.

**Table 7:** Total concentration of each chemical in GNRs, reported oral LOD50 for each chemical and the highest i.v. (intervenes) dose of GNRs tested in for rat model.

Chemical Name	Total concentration in Sona Nanotech Inc.™ GNRs solution	Oral LD50 (Rat) Values	Highest tolerated dose of Sona Nanotech Inc.™ GNRs tested in Rat model
PEG	45-65mg/L	>4 g/Kg^1^	5mL/Kg
Surfactant 1	20-35mg/L	71 mg/kg	
Surfactant 2	60-72mg/L	410 mg/kg^2^	

^1^Thiol PEG are reported to have variable LOD and 4g/kg is the lowest reported value.

^2^LOD value for surfactant 2 is unavailable and reported value is for CTAB.

The LD50 of the GNRs used in this study was not directly calculated; however, comparable products demonstrated no mortality in mice following a single intravenous administration at doses up to 1,000 mg/kg (35). Histological analysis in these studies revealed significant GNP accumulation within the liver and spleen, along with the formation of microgranulomas in the liver. Despite this accumulation, serum biochemical profiles indicated no overt signs of metabolic, renal, or hepatic dysfunction (35). By comparison, the i.t. dose used in the present study was significantly lower, at 200 µg per tumor (~10 mg/kg), representing only 1% of the maximum dose evaluated in the study by Bahamonde et al.

Compared to intravenous administration, intratumoral injection is expected to result in even lower systemic toxicity due to localized delivery and reduced nanoparticle distribution to off-target organs such as the liver and spleen. This localized approach minimizes systemic exposure, further enhancing the safety profile of the treatment. Therefore, we conclude that the concentration used in our study is far below any lethal dose. Additionally, no weight loss or signs of toxicity were observed in the mice during the course of our study, confirming that the treatment was well tolerated.

Endotoxin levels in GNR preparations were quantified and confirmed to be below the threshold limit of 4.66 endotoxin units per milligram of gold (EU/mg Au) (**Table 8**), meeting safety standards for *in vivo* applications (USP-BET85). These findings suggest Sona Nanotech Inc.™’s GNR formulations are biochemically suitable and safe for therapeutic use.

**Table 8:** Measured and estimated endotoxin levels for GNRs.

Sample code	Turbidity, EU/mg Au (% spike recovery)	The calculated endotoxin limit
Sample 1	< 0.5 (61)	4.66 EU/mg Au
Sample 2	< 0.5 (89)	

### GNR activation by NIR is required to generate THT

**Figure 2:**
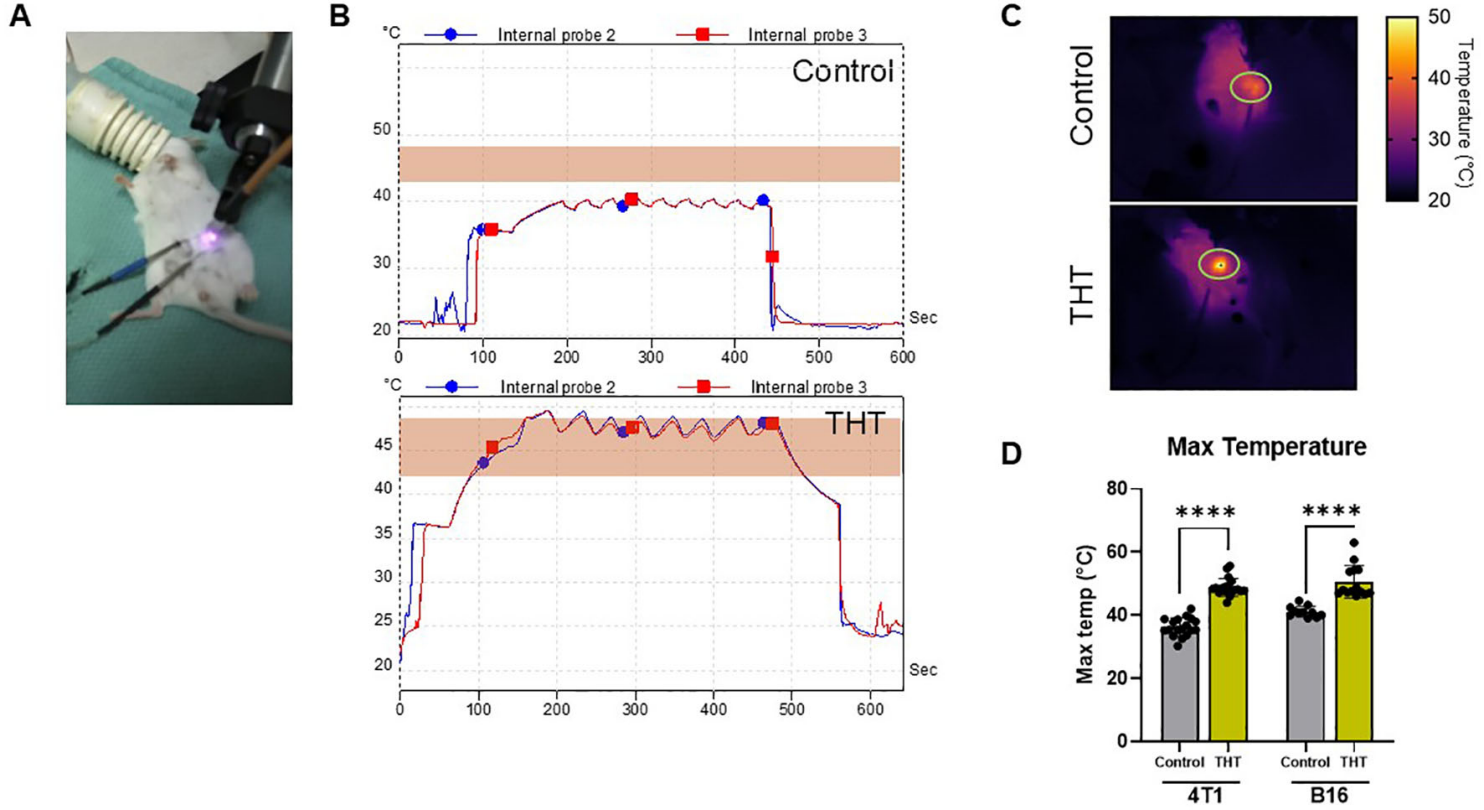
GNR-Enhanced Laser Irradiation Induces Tumor THT, Absent in Laser-Only Controls. **(A)** Photo of the experimental setup, showing a BALB/c mouse bearing a 4T1 tumor, with two internal temperature probes used to monitor the effects of THT versus controls. **(B)** Representative temperature profiles of 4T1 tumors in BALB/c mice generated from internal probes during NIR light, comparing the temperature changes in GNR-injected tumors to those in laser-only controls. **(C, D)** Quantitative analysis of surface temperatures in BALB/c mice with 4T1 tumors (n=18 control, 19 THT) and C57BL/6 mice with B16-F10 tumors (n=11 control, 14 THT). **** P ≤ 0.0001.

To confirm the efficacy of GNRs to create THT, we compared temperature profiles between GNR-injected tumors and controls exposed to NIR light. An example setup utilizing a BALB/c mouse with a 4T1 tumor and two internal temperature probes is illustrated in **Figure 2A**. GNR-injected 4T1 tumors exhibited a rapid and significant temperature increase upon NIR light exposure, reaching a peak internal temperature of 48°C within 60-100 seconds. This hyperthermic state was maintained on average for 20 seconds post-exposure, demonstrating the effectiveness of GNRs in facilitating thermal conversion. Subsequent NIR light stimulations required only 20 seconds to achieve a surface temperature of 48°C, and this temperature (range 42-48°C) was sustained for 5 minutes (**Figure 2B**). In contrast, tumors in the NIR light-only control group exposed to the same intensity and duration of NIR light exhibited minimal temperature elevation, always remaining below 42°C, insufficient to trigger apoptotic cell death (**Figure 2B**).

Quantitative analysis of surface temperatures revealed that in 4T1 tumors, GNR-treated mice achieved a maximum surface temperature of 48.92°C ( ± 2.8°C), compared to 36.32°C ( ± 2.89°C) in controls (**Figures 2C, D**). Similarly, in B16-F10 tumors, GNR-treated mice reached a maximum temperature of 50.65°C ( ± 5.13°C), while controls reached only 41.17°C ( ± 1.69°C) (**Figure 2D**) (36). These results confirm that GNRs are required and responsible for the moderate THT related to NIR light exposure in tumors and validate their potential for the precise application of THT in cancer treatment.

### THT related tumor responses, associated with innate immune activation and ICD are observed within 48 hours following treatment with THT to the tumor microenvironment

**Figure 3:**
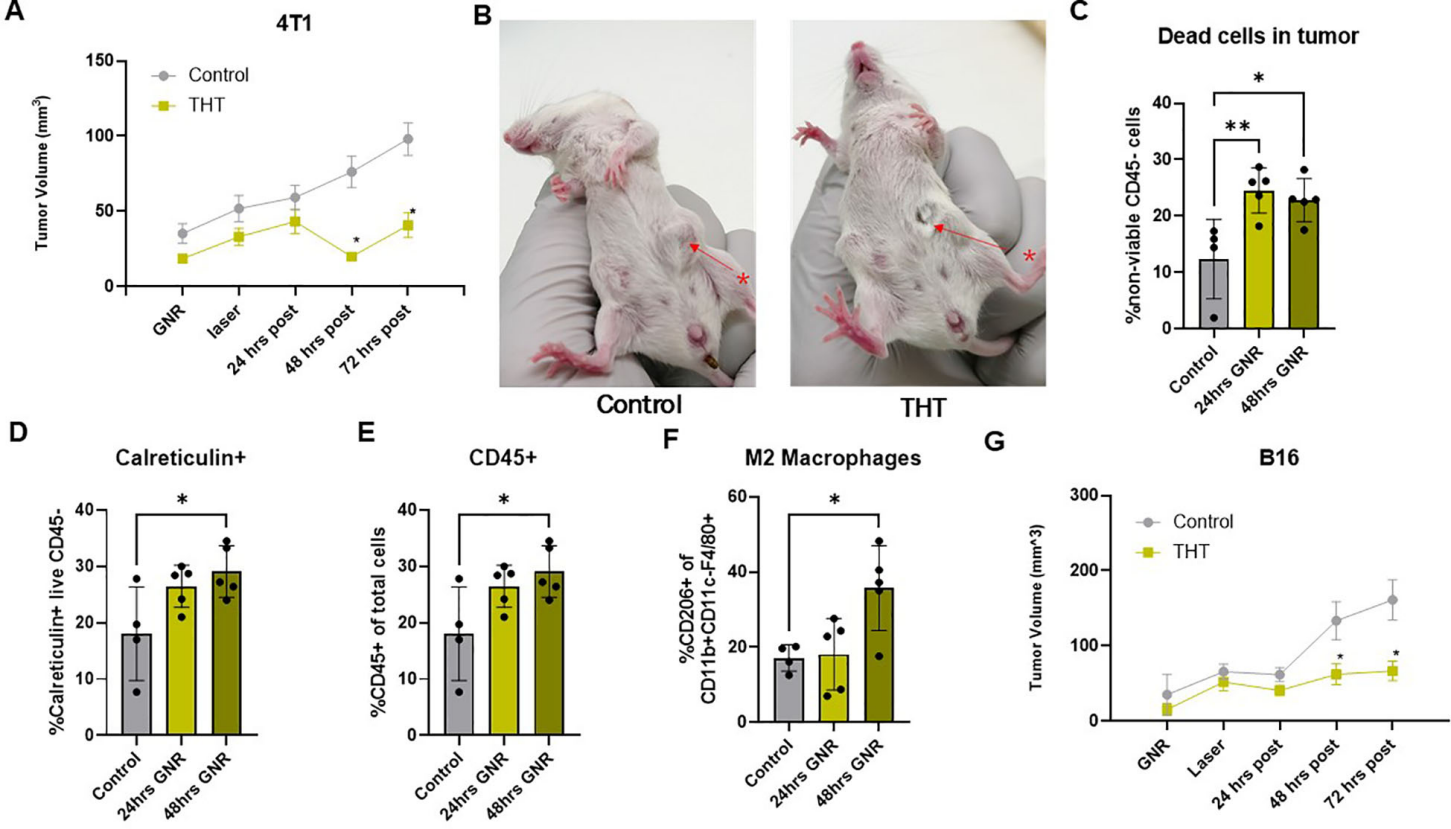
GNR-Mediated THT Induces Tumor Shrinkage, Cell Death, and Immune Activation in 4T1 Models and Growth Suppression in B16-F10 Models Within 48 Hours Post-Treatment. **(A)** Tumor volumes in 4T1 tumor models following GNR-mediated THT treatment (n=24 control, n=28 THT), error bars represent SEM. **(B)** Representative images of a control mouse and a THT-treated mouse 48 hours post-irradiation, alongside a comparison of tumor volumes between GNR-treated and control groups. **(C)** Flow cytometry analysis of cell viability (as determined by BD Horizon™ Fixable Viability Stain 510) in all cells of a 4T1 tumors 24 and 48 hours post-THT (n=5). **(D)** Levels of extracellular calreticulin in 4T1 tumors (CD45-) 24 and 48 hours post-THT (n=5). **(E)** Flow cytometry data showing the percentage of CD45^+^ immune cells in 4T1 tumors 24 and 48 hours post-treatment (n=5). **(F)** Analysis of M2 macrophage levels (gated on CD45+/CD11b+F480+CD206+) in 4T1 tumors 24 and 48 hours post-THT (n=5). **(G)** Tumor volume measurements in the B16-F10 model within 48 hours post-laser treatment (n=19 control, n=23 THT), error bars represent SEM. *p < 0.05, **p <0.01.

To evaluate the therapeutic effects of a single treatment with THT on tumor progression, we measured tumor volumes in 4T1 and B16-F10 tumor models 24, 48, and 72 hours post-NIR light exposure. In the 4T1 tumor model, significant tumor reduction was observed in the THT treated group within 48 hours (**Figure 3A**). Tumor volume decreased to 19.9 mm³ ( ± 15.6 mm³) 48 hours post- NIR activation, down from 43.1 mm³ ( ± 42.9 mm³) at 24 hours post-NIR activation. In contrast, the control group showed an increase in tumor volume, with measurements increasing to 76.2 mm³ ( ± 51.2 mm³) 48 hours post-NIR exposure , up from 59.2 mm³ ( ± 42.0 mm³) 24 hours post-NIR activation. This reduction was statistically significant at both 48- and 72-hours post-NIR light activation (**Figure 3B**). A representative photo of a control mouse and THT treated mouse 48 hours post-NIR exposure is shown in **Figure 3B**.

Flow cytometry analysis (**Supplementary Figures 1A, C**) demonstrated significantly elevated levels of non-viable CD45- cells in 4T1 tumors treated with THT at 24- and 48-hours post-treatment compared to controls. This increase in cell death aligns with the observed tumor volume reduction and underscores the cytotoxic effects of THT. At 48 hours post-THT, expression of calreticulin, a peptide-binding heat shock protein (HSP), on the cell membrane of CD45- cells was significantly increased (**Figure 3D**). This suggests the induction of ICD, further highlighting the potential for GNR-mediated THT to not only directly kill tumor cells but also to stimulate an immune response (37).

A non-significant trend of increased annexin V on CD45- cells (**Supplementary Figure 1B**) suggests enhanced apoptosis. Moreover, THT-treated 4T1 tumors showed a significantly higher percentage of infiltrating CD45+ immune cells at 48 hours post-treatment compared to controls (**Figure 3E**). Additionally, M2 macrophage levels were significantly elevated in THT-treated tumors at 48 hours post-treatment (**Figure 3F**). **Supplementary Figure 3C** indicates a modest increase in non-viable CD45+ cells (6.96 ± 1.789% at 24 hours), which was notably lower than CD45- cell death, highlighting THT's preferential targeting of CD45- cells. Transient decreases in dendritic cells (DCs, CD11b+CD11c+) and MHCII+ DCs were observed at 24 hours but recovered by 48 hours (**Supplementary Figures 1D, E**). Trends of reduced total macrophages, M1 macrophages (**Supplementary Figures 1F, G**), and the M1/M2 ratio (**Supplementary Figure 1H**) were observed post-THT, though these changes were not statistically significant.

Similarly, following THT, tumor volumes in the B16-F10 tumor model were significantly smaller (62.0 mm³ ± 68.1 mm³, 66.6 mm³ ± 60.6 mm³) compared to controls (133.3 mm³ ± 110.2 mm³, 161.1³ ± 116.1 mm³) 48- and 72-hours post-NIR light activation, respectively (**Figure 2G**). These findings indicate that THT as a single modality effectively shrinks tumors and activates innate immunity in multiple preclinical models.

### Single exposure to THT induces innate immune responses but does not sustain long-term tumor control

**Figure 4:**
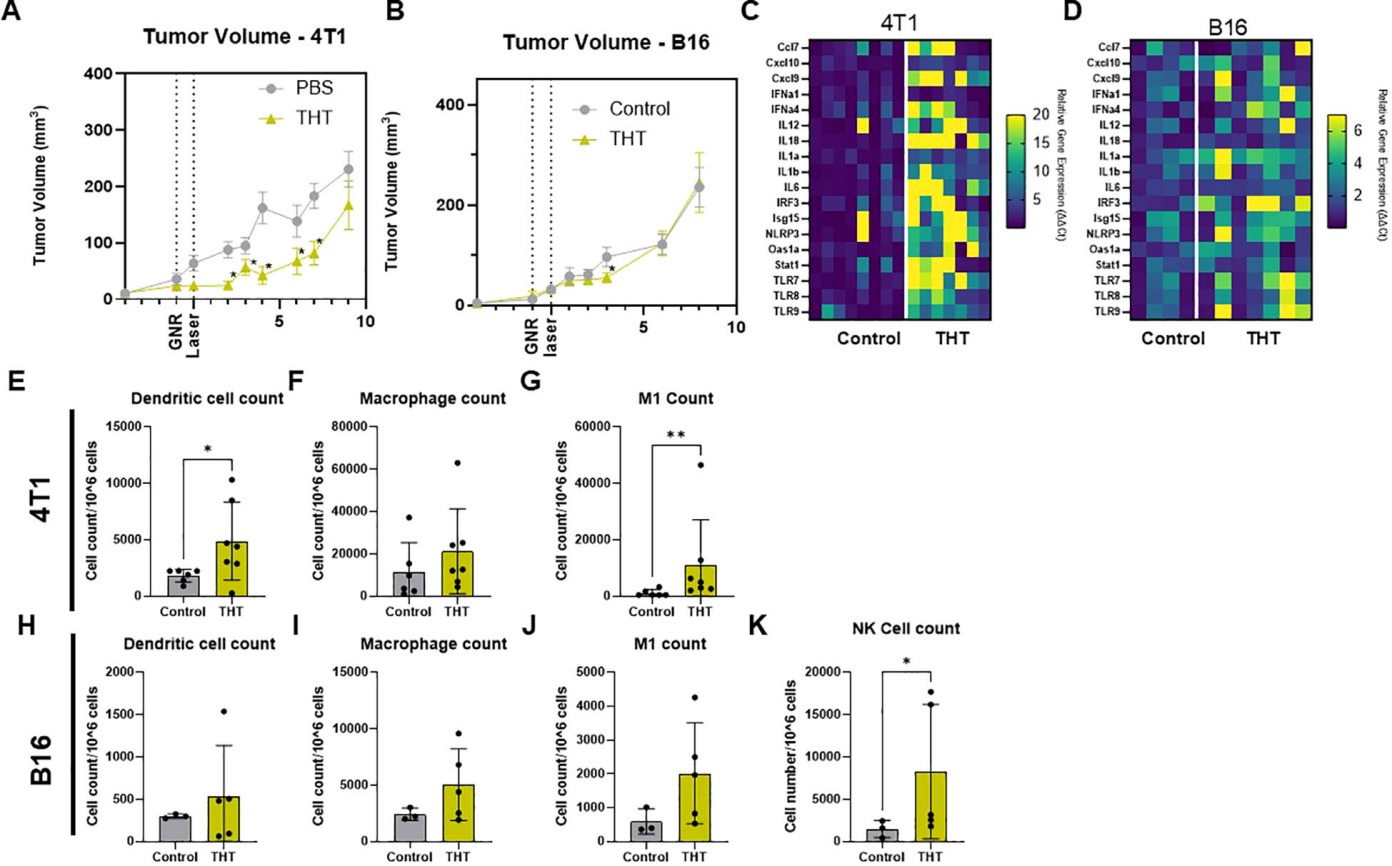
GNR-Induced THT Results in Tumor Regrowth After Initial Reduction, Despite Upregulation of STING Pathway Genes and Increased Innate Immune Cell Levels. **(A)** Tumor volume measurements in 4T1 models following THT treatment compared to control (n=16 control, n=15 THT), error bars represent SEM. **(B)** Tumor volume analysis in B16-F10 models (n= 9 control, n=11 THT), error bars represent SEM. **(C, D)** Gene expression analysis 8 days post-laser treatment showing upregulation of STING pathway genes in 4T1 (n= 8 control, 7 THT) and B16-F10 tumors (n = 4 control, 7 THT) subjected to THT or control. **(E-G)** Flow cytometry analysis of dendritic cells (CD45+CD11b+F4/80-CD11c+MHCII+), M1 macrophages (CD45+CD11b+F4/80+CD80+CD86+), and macrophages (CD45+CD11b+F4/80+) in 4T1 tumors post-THT, (n=6 control, n= 7 THT). **(H-K)** Flow cytometry analysis of dendritic cells, M1 macrophages, macrophages and NK cells (CD3- NK1.1+) in B16-F10 tumors post-THT, (n=3 control, n= 5 THT). *p < 0.05, **p <0.01.

Following the initial response to a single THT treatment, tumor regrowth was observed in both 4T1 and B16-F10 models over time. In the 4T1 model, THT led to a significant reduction in tumor size within the first 48 hours post-NIR activation. However, tumor volumes began increasing by day 6. By day 9, tumors had regrown to volumes comparable to control (**Figure 4A**). Similarly, in the B16-F10 model, THT-treated tumors showed significant reductions 3 days post-treatment, but tumor regrowth to control volumes was evident by day 6 (**Figure 4B**). These results indicate that a single exposure to THT is insufficient to sustain long-term tumor volume control. Notably, a double THT regimen, where a second NIR activation was applied at 4 (4T1) or 7 (B16-F10) days after the initial treatment, was sufficient to heat the tumor to hyperthermic temperatures for 5 mins and provided a slight but non-significant reduction in tumor regrowth compared to single THT treatment, suggesting limited therapeutic benefit from repeated exposure under these conditions (**Supplementary Figures 2A, B**). Interestingly, analysis of gene expression 8 days post-NIR light exposure revealed a significant upregulation of genes involved in the STING (Stimulator of Interferon Genes) cGAS (cyclic GMP-AMP synthase) pathway in tumors subjected to THT in both models, as illustrated in **Figures 4C** (4T1) and 4D (B16-F10). This upregulation suggests enhanced recognition of tumor-associated antigens and a subsequent activation of immune signaling (38). The increased expression of STING cGAS pathway genes indicates that THT induces tumor cell death while triggering an innate immune response.

Flow cytometry analysis further showed that THT increased innate immune cell populations in both 4T1 and B16-F10 models, though the specific immune responses varied. In 4T1 tumors, there was a significant increase in dendritic cells (**Figure 4E**), M1 macrophages (**Figure 4G**), and a trend toward higher overall macrophage counts (**Figures 4G**). B16-F10 tumors exhibited no significant changes in dendritic cells (**Figure 4H**) but did trends toward increased macrophage and M1 macrophage levels (**Figures 4I, J**), and a significant increase in NK cells (**Figure 4K**). These findings highlight the complexity of THT-induced immune responses, which vary depending on the tumor model.

### I.t. IL-2 has no effect on naïve tumors in 4T1 and B16-F10 models, but it enables sustained immune responses preventing tumor regrowth following a single treatment with THT

While a single treatment with THT effectively reduced tumor volume and stimulated host innate immunity, tumor regrowth was observed over time. We hypothesized that this regrowth may be partially related to significantly more M2 macrophage in the TME observed two days post THT, potentially enabling further tumor growth. In consideration of these observations, we sought to further enable the THT-initiated immune response with the addition of i.t. IL-2.

**Figure 5:**
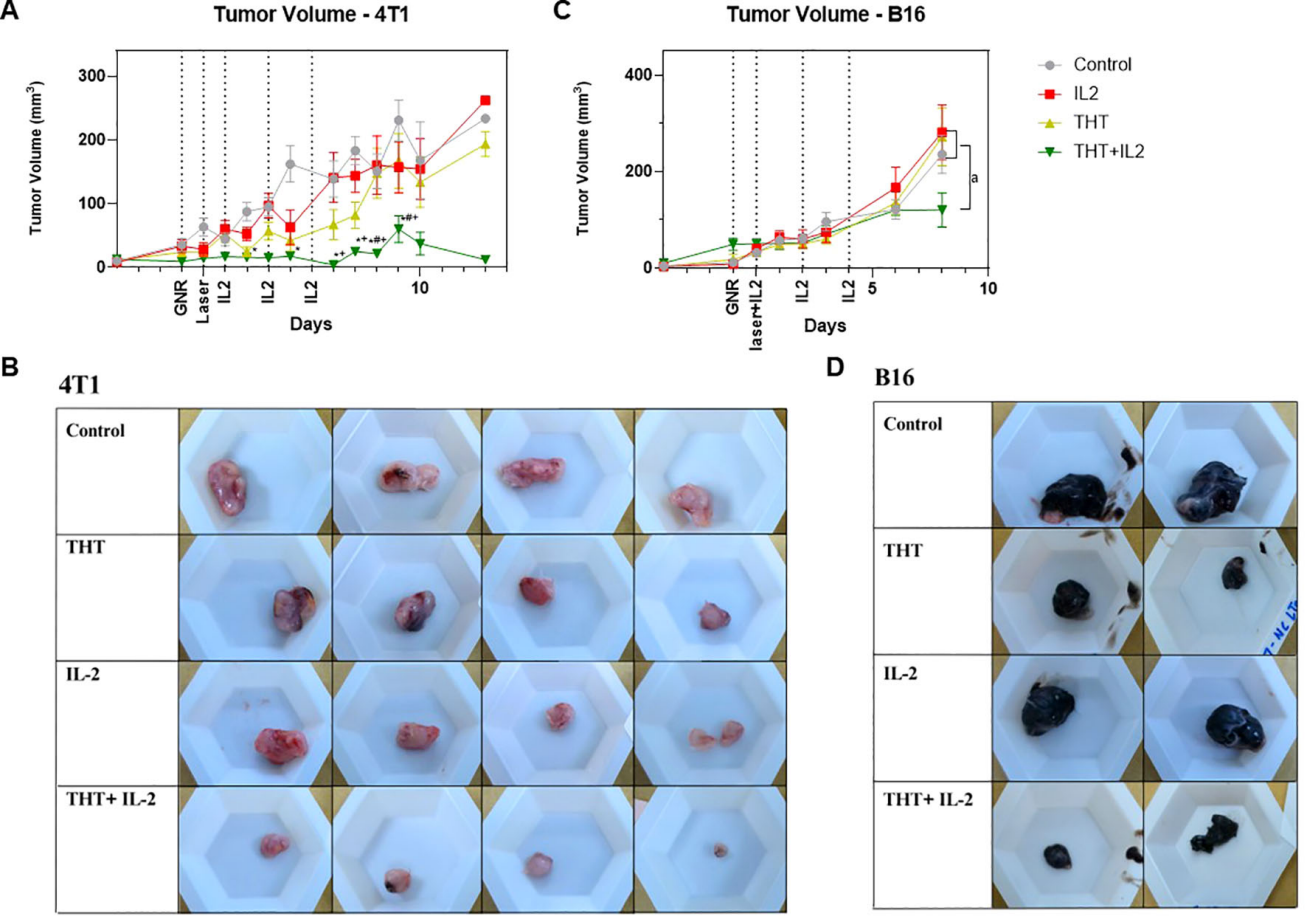
I.t. IL-2 Treatments Prevent Tumor Regrowth Following GNR-Induced THT. **(A)** Tumor volume measurements over a 14-day period post-laser treatment for each group: THT alone, i.t. IL-2 alone, THT plus i.t. IL-2, and control in the 4T1 model (n=16 control, 14 IL2, 15 THT, 12 THT+IL2), error bars represent SEM. **(B)** Representative images of extracted 4T1 tumor **(C)** Tumor volume measurements up to day 8 post-laser treatment in the B16-F10 model, showing the comparison between the same groups (n=9 control, 7 IL2, 10 THT, 10 THT+IL2), error bars represent SEM. **(D)** Representative images of extracted B16-F10 tumor. *p < 0.05 *vs*. control, ^+^p < 0.05 *vs*. IL-2, ^#^p < 0.05 *vs*. THT (ANOVA), ^a^p < 0.05 (t-test).

IL-2 is known for its ability to activate and expand T-cells (39). Mice bearing 4T1 or B16-F10 tumors were subjected to THT, followed by a series of i.t. IL-2 injections (THT plus i.t. IL-2). In the 4T1 model, the combination of THT plus i.t. IL-2 injections resulted in sustained tumor regression, with no tumor regrowth observed throughout the 14-day monitoring period post- NIR activation. Tumor volumes in the THT plus i.t. IL-2 treated group were significantly reduced compared to controls 2 days following THT treatment, significantly reduced compared to the i.t. IL-2 only group 6 days post-NIR exposure, and significantly reduced compared to THT alone 8 days post-NIR exposure. The THT plus i.t. IL-2 group maintained a low average tumor volume, peaking at 60.1 mm³ (± 55.7 mm³) on day 9 post THT, compared to 230 mm³ (± 96.1 mm³) in the control group, 157.1 mm³ (± 112.7 mm³) in the i.t. IL-2 only group, and 166.9 mm³ (± 148.8 mm³) in the THT only group (**Figure 5A**). Similarly, final tumor weights of THT plus i.t. IL-2 treated tumors were significantly reduced compared to control and i.t. IL-2 treated tumors (**Figure 5B**, **Supplementary Figure 3A**).

In the B16-F10 model, similar trends were observed. Eight days post-NIR exposure, the average tumor volume in the THT plus i.t. IL-2 group [120.7 mm³ (± 29.6 mm³)] was significantly lower compared to 235.7 mm³ (± 111.1 mm³) in the control group, 281.6 mm³ (± 150.5 mm³) in the i.t. IL-2 only group, and 271.5 mm³ (± 169.8 mm³) in the THT only group (**Figure 5C**). The final tumor weights of THT plus i.t. IL-2-treated tumors showed a reduction compared to controls, though the decrease was not statistically significant (**Figure 5D**, **Supplementary Figure 3B**). Mouse weights did not change significantly during the course of these experiments (**Supplementary Figures 3C, D**).

These results demonstrate that i.t. IL-2 injections effectively prevent tumor regrowth following THT. This approach offers a promising strategy for enhancing the long-term efficacy of THT-based cancer treatments.

### I.t. IL-2 treatment enhances CD8+ T cell infiltration, central memory differentiation, and PD-1 expression in tumors following NIR-induced THT

**Figure 6:**
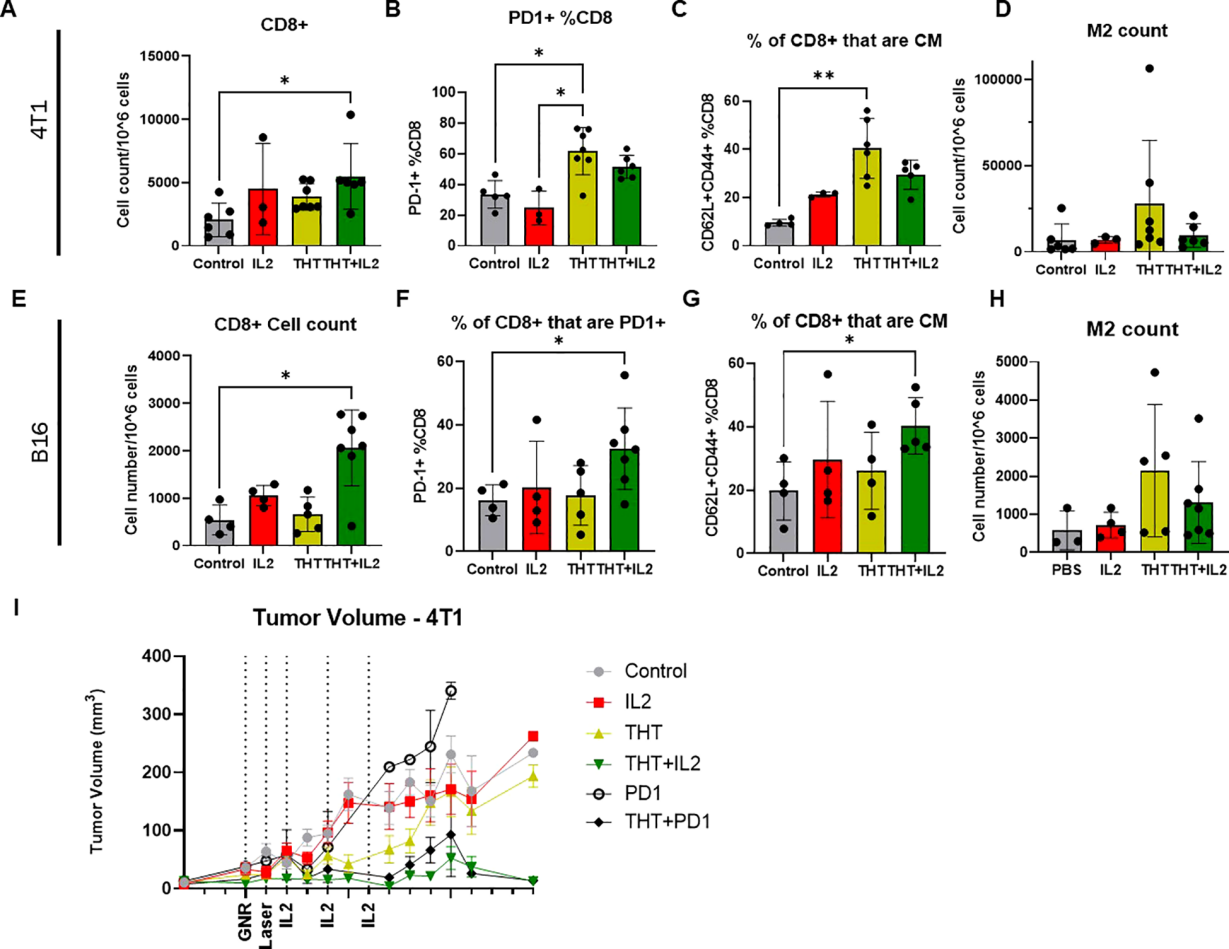
IL-2 Treatment Enhances CD8+ T Cell Infiltration, Central Memory Differentiation, and PD-1 Expression in Tumors Following GNR-Induced THT. Panel **(A)** shows the quantification of CD8+ T cells (gated on CD3+CD8+) in 4T1 tumors across different treatment groups (n= 6 Control, 3 IL2, 7 THT, 6 THT+IL2). Panel **(B)** illustrates the expression of PD-1+ on CD8+ T cells (gated on PD-1+CD8+CD3+) in 4T1 tumors. Panel **(C)** displays the frequency of CD8+ central memory (CM) T cells (gated on CD62L+CD44+CD8+CD3+) in 4T1 tumors. Panel **(D)** presents the quantification of M2 macrophages (gated on CD45+/CD11b+F480+CD206+) in 4T1 tumors. In the B16-F10 model, panel **(E)** quantifies CD8+ T cells (gated on CD3+CD8+) across different treatment groups (n= 4 Control, 4 IL2, 5 THT, 7 THT+IL2). Panel **(F)** shows the expression of PD-1+ on CD8+ T cells (gated on PD-1+CD8+CD3+) in B16-F10 tumors. Panel **(G)** illustrates the frequency of CD8+ central memory (CM) T cells (gated on CD62L+CD44+CD8+CD3+) in B16-F10 tumors, while panel **(H)** quantifies M2 macrophages (gated on CD45+/CD11b+F480+CD206+) in B16-F10 tumors. **(I)** Tumor volume measurements over a 14-day period post-laser treatment for each group: THT alone, i.t. IL-2 alone, THT plus i.t. IL-2, and control in the 4T1 model (n=16 control, 14 IL2, 15 THT, 12 THT+IL2, 2 PD1, 4 PD1+THT), error bars represent SEM. *p<0.05, **p <0.01.

I.t. IL-2 administered following THT significantly increased CD8+ T cell infiltration into the TME. Flow cytometry analysis, as detailed by the gating scheme in **Supplementary Figure 4A**, showed a substantial increase in CD8+ T cell numbers in tumors treated with THT plus i.t. IL-2 compared to THT alone at the study endpoint (Day 8–14). In both the 4T1 (**Figures 6A–D**) and B16-F10 models (**Figures 6E-H**), CD8+ T cell infiltration was significantly higher in the THT plus i.t. IL-2 groups compared to controls (**Figures 6A, E**).

In 4T1 tumors, CD3+ and CD4+ T cells trended higher in the THT plus i.t. IL-2 group, though these changes were not statistically significant (**Supplementary Figures 5B, C**). Regulatory T cells (Tregs; CD4+FoxP3+CD25+) were significantly elevated in the THT plus i.t. IL-2 group compared to THT alone (**Supplementary Figure 4D**), consistent with previous reports of IL-2-induced Treg expansion (40). PD-1 expression on CD4+ T cells was higher in the THT plus i.t. IL-2 group compared to i.t. IL-2 alone, with a non-significant increase compared to PBS-treated controls (**Supplementary Figure 4E**). This is consistent with the known promotion by hyperthermia of increased coinhibitory molecule expression on immune cells (23). For antigen-presenting cells, dendritic cells (DCs, CD11c+MHCII+) and macrophage populations remained unchanged (**Supplementary Figures 4F, G**). However, M1 macrophages were significantly elevated in THT-treated groups relative to controls, indicating a pro-inflammatory shift (**Supplementary Figure 4H**). Representative immunohistology stains for CD3+ T cells further support increased infiltration in THT plus i.t. IL-2-treated tumors (**Supplementary Figures 4I**), demonstrating significantly enhanced immune cell presence compared to other treatment groups. Notably, while no CD3+ infiltration is observed in control tumors, staining reveals moderate infiltration in tumors treated with IL-2 or THT alone. The highest levels of CD3+ T cell infiltration are evident in tumors receiving the combined THT and IL-2 treatment, aligning closely with our flow cytometry findings and highlighting the synergistic effects of this therapeutic combination.

In the B16-F10 model, CD3+ T cells were significantly higher in the THT plus i.t. IL-2 group compared to controls (**Supplementary Figure 5A**). CD4+ T cells were elevated in the i.t. IL-2 group compared to THT alone. Tregs were increased in both the i.t. IL-2 and THT plus i.t. IL-2 groups compared to both the PBS control and THT alone groups, though these changes were not statistically significant (**Supplementary Figures 5B, C**). PD-1 expression on CD4+ T cells trended higher in the THT plus i.t. IL-2 group but was not significant (**Supplementary Figure 5D**). Similarly, no significant changes were observed in DCs, macrophages, or M1 macrophages (**Supplementary Figures 5E-G**). Notably, NK cells were elevated in all treatment groups, with a significant increase in the i.t. IL-2 group (**Supplementary Figure 5H**).

In 4T1 tumors, THT alone significantly increased the percentage of PD-1+ CD8+ T cells, while the addition of i.t. IL-2 did not further increase PD-1+ CD8+ T cells beyond THT alone (**Figure 6B**). In the B16-F10 model, THT plus i.t. IL-2 resulted in significantly higher PD-1 expression on CD8+ T cells compared to PBS controls and THT alone (**Figure 6F**), indicating different PD-1 induction mechanisms in 4T1 and B16-F10 tumors.

THT alone significantly increased CD8+ central memory (CM) cells in the 4T1 model (**Figure 6C**). Although THT plus i.t. IL-2 trended toward further increases in CM cells, these differences were not statistically significant. In the B16-F10 model, THT plus i.t. IL-2 significantly elevated CM cell levels compared to controls (**Figure 6G**). These findings are consistent with IL-2’s role in regulating CD8+ T cell biology, promoting effector differentiation and the formation of long-lived memory cells (41).

To further investigate increased PD-1 expression on CD8+ T cells, we evaluated the impact of anti-PD-1 monotherapy and its combination with THT on 4T1 tumor growth. While anti-PD-1 monotherapy did not significantly affect tumor growth compared to controls, combining anti-PD-1 with THT significantly reduced tumor volume, yielding results comparable to THT plus i.t. IL-2 (**Figure 6I**). This aligns with preclinical data suggesting that combining hyperthermia with immune checkpoint inhibitors, particularly anti-PD-1, enhances tumor antigen-specific T cell responses and promotes lasting tumor immunity (23).

In summary, i.t. IL-2 administration following GNR-induced THT significantly enhances CD8+ T cell infiltration in both tumor models. While IL-2 promotes central memory cell differentiation and increases PD-1 expression in the B16-F10 model, THT alone is sufficient to induce central memory cell differentiation and PD-1 expression in the 4T1 model. Furthermore, the combination of i.t. IL-2 with THT mitigates the increase in immunosuppressive M2 macrophages observed with THT alone, underscoring its potential to counteract tumor-promoting immune cells. These findings highlight the therapeutic benefits of combining i.t. IL-2 with hyperthermia-based treatments to modulate the immune response and enhance cancer treatment outcomes.

### GNR-induced THT combined with i.t. IL-2 reduces contralateral 4T1 tumor size and enhances CD8+ T cell infiltration

**Figure 7:**
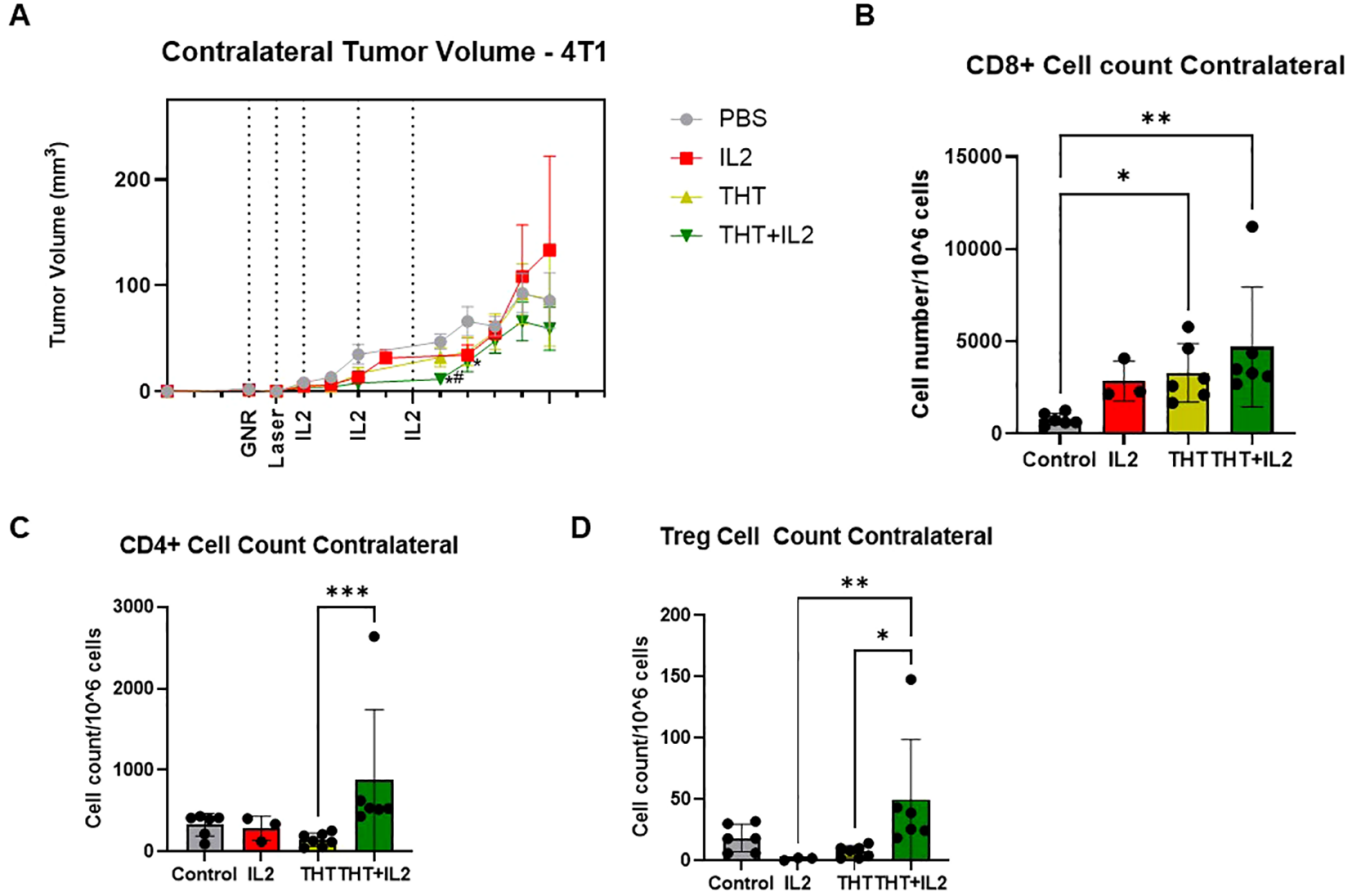
GNR-Induced THT Combined with IL-2 Reduces Contralateral 4T1 Tumor Size and Enhances CD8+ T Cell Infiltration. **(A)** shows the tumor volume of contralateral (untreated) 4T1 tumors in mice with bilateral tumors (n= 8 Control, 6 IL2, 8 THT, 7 THT+IL2). **(B)** quantifies CD8+ T cells (gated on CD3+CD8+) in contralateral tumors. **(C)** CD4+ T cells in contralateral tumors. **(D)** Treg (CD3+CD4+FoxP3+CD25+) levels in contralateral tumors, (for all flow analysis: n= 6 Control, 3 IL2, 6 THT, 6 THT+IL2). *p<0.05 *vs*. control, ^#^p<0.05 *vs*. THT. **p<0.01, ***p<0.001.

To assess the systemic effects of combining GNR-induced THT with i.t. IL-2 injections, we evaluated the impact on contralateral (untreated) 4T1 tumors in mice with bilateral tumors. The combination treatment resulted in a significant reduction in contralateral tumor volume compared to controls on days 6 and 7 post-NIR exposure, and a significant reduction in tumor volume observed on day 6 post-NIR exposure in the combination group compared to the THT alone group (**Figure 7A**). Flow cytometry revealed a significant increase in CD8+ T cells within the contralateral tumors of both THT and THT plus i.t. IL-2-treated mice (**Figure 7B**). Additionally, **Figure 7C** shows that THT plus i.t. IL-2 significantly increased CD4+ cells in contralateral tumors compared to THT alone, while **Figure 7D** demonstrates that Tregs were significantly elevated in the THT plus i.t. IL-2 group compared to controls. **Supplementary Figure 6A** shows no significant differences in DCs between groups, and **Supplementary Figure 6B** indicates no significant differences in macrophage populations between groups. These findings suggest that GNR-induced THT not only enhances local tumor immunity but also induces a systemic adaptive immune response that extends beyond the primary treatment site.

## Discussion

In this study, we aimed to establish proof of concept using two distinct, widely accepted preclinical cancer models to demonstrate that hyperthermia can be precisely, reliably, and effectively delivered to tumors, minimizing side effects. The use of gold-based nanoparticles as catalysts for photothermal conversion, enabling hyperthermia, is well-documented (8, 42). Among these, GNRs offer distinct advantages over other gold nanoparticle shapes, such as nanospheres or nanoshells. These include tunable optical properties in the NIR range, higher photothermal conversion efficiency, and a favorable aspect ratio for heat generation (42). These characteristics make GNRs particularly suitable for precise thermal modulation of the TME. Emerging evidence indicates that hyperthermia can enhance immunotherapy by promoting immune cell infiltration and improving antigen presentation (12). To our knowledge, this is the first study to specifically combine hyperthermia with i.t. IL-2 injections, offering a novel strategy to stimulate anti-tumor immunity. Additionally, most studies on GNP-induced hyperthermia rely on systemic administration of nanoparticles, using tumor neo-vasculature for preferential deposition (43). To minimize systemic exposure and enhance nanoparticle concentration at the tumor site, we employed direct i.t. injection of GNRs. Specifically, we utilized Sona Nanotech Inc.^TM^'s GNRs, which are biocompatible, toxin-free, and activated by 860 nm NIR light, enabling controllable mild hyperthermia.

Our data demonstrate that Sona Nanotech Inc.^TM^’s GNR induced hyperthermia can rapidly (within 100 s) achieve therapeutic temperatures sufficient to induce tumor cell apoptosis (**Figure 2**), which aligns with other GNR literature (44–46). This contrasts with more time consuming, invasive, and ablative forms of inducing tumoral hyperthermia, such as isolated hyperthermic chemoperfusion (2) or those with less direct tumor targeting abilities such as ultrasound or electromagnetic heating (47). Additionally, while other GNR systems reported in the literature required extended heating protocols, such as four 15-minute irradiation sessions, Sona Nanotech Inc.™ GNRs achieved comparable tumor cell death outcomes with a single 5-minute NIR light activation (**Figure 3**) (46). Notably, adding a second NIR light exposure provided only marginal additional benefits (**Supplementary Figures 4A, B**).

Our study also functioned to further validate the critical role of THT in inducing ICD, evidenced by elevated calreticulin levels in THT-treated 4T1 tumors (**Figure 3D**), a well-established marker of ICD. THT-induced ICD functions to stimulate innate immune responses through mechanisms involving reactive oxygen species (ROS), endoplasmic reticulum (ER) stress, and enhanced presentation of tumor neoantigens. These processes collectively generate "enabler" and "eat me" signals that recruit immune cells into the TME, thereby fostering a more pro-inflammatory and favorable anti-cancer immune response (23, 48–54). This was particularly impressive given that both 4T1 and B16-F10 tumors are classified as immunogenically "cold" tumors, which are typically resistant to immune-mediated therapies (55, 56). Further confirmation of enhanced innate immunity post-THT is provided by upregulation of the STING-cGAS immune pathway in 4T1 tumors treated with THT (**Figure 4**), along with increased immune cell infiltration (**Figure 3E**). The STING cGAS pathway plays a crucial role in immune activation by recognizing cytosolic DNA, including mitochondrial DNA (mtDNA), which triggers inflammation and DNA damage, key for initiating an immune response against tumors (57, 58).

Interestingly, following THT, an increase in classically immunosuppressive M2 macrophages was observed (**Figure 3F**), which aligns with studies showing fever-range hyperthermia induces M2-like polarization in macrophage cell lines (53). M2 macrophages promote tumor progression through immune suppression, secretion of anti-inflammatory cytokines like IL-10 and TGF-β, and factors supporting angiogenesis and extracellular matrix remodeling. The increase in M2 macrophages reflects a complex interplay between pro-inflammatory and immunosuppressive responses within the TME, potentially contributing to tumor regrowth (**Figures 3A, G**) (59). Future studies combining THT with therapies targeting M2 macrophage polarization could provide insights into mechanisms underlying tumor regrowth.

Tumor regrowth after hyperthermic treatment has not been extensively studied, but hyperthermia has been shown to induce immunosuppressive pathways, such as increased expression of IL-10, which dampens immune responses and promotes tumor tolerance (60). Based on these observations, we hypothesized that the addition of i.t. IL-2 to THT would help shift the balance toward a more pro-inflammatory and anti-tumor TME. IL-2 promotes the activation and expansion of effector T cells and NK cells, driving anti-tumor immunity. It also counteracts immunosuppressive macrophages by promoting the differentiation of pro-inflammatory M1 macrophages, which produce cytokines like TNF-α and IL-12 that enhance tumor-killing immunity (61). Therefore, combining THT with i.t. IL-2 may overcome M2 macrophage-mediated immunosuppression and boost anti-tumor responses. Our study shows that i.t. IL-2 therapy alone has minimal effects on 4T1 and B16-F10 tumors, consistent with the challenges of treating **“**cold**”** tumors with immunotherapies (**Figures 4B**, **5A**) (62). Interestingly, we observed a slight increase in tumor volume in tumors treated with i.t. IL-2, which was unexpected. However, this could be attributed to pseudoprogression, likely caused by increased infiltration of immune cells compared to the control group (**Figure 6A**, **Supplementary Figure 4I**) (63). As expected and consistent with our hypothesis that ICD must first be initiated to enhance the efficacy of immunotherapies, the combination of THT and i.t. IL-2 induced sustained tumor regression (**Figures 5A, B**) and increased CD8+ T cell infiltration in the TME at the study endpoint (**Figure 6A**). However, as documented in the literature, IL-2 therapy was found to increase Treg levels (**Supplementary Figures 4D**, **5C**), which are known to suppress anti-tumor immunity and may limit the therapeutic effect of IL-2 (61). Based on this finding, it’s possible that this treatment regimen could be further optimized to improve anti-tumor immunity, perhaps through the use of an IL-2 mutant “superkine” that preferentially binds and activates cytotoxic lymphocytes (CTLs) and NK cells (64), or a different interleukin, such as IL-15, that preferentially promotes the proliferation and activation of CTLs and effector T cells in comparison to Tregs (65).

Additional analysis of immune cell infiltration in tumors following THT in the 4T1 and B16-F10 models revealed that infiltrating CD8+ T cells expressed PD-1 receptors (**Figures 6B, F**). This led us to hypothesize that combining THT with a PD-1 inhibitor could further enhance the anti-cancer immune response. PD-1, expressed on activated T cells, inhibits T cell activation when bound to its ligand PD-L1, allowing tumors to evade immune surveillance (66). Notably, 4T1 tumors, which were previously unresponsive to PD-1 therapy, became responsive after THT treatment (**Figure 6I**). Similar to our findings with i.t. IL-2, THT followed by systemic anti-PD-1 therapy resulted in prolonged tumor regression (**Figure 6I**). These results further support that THT induced ICD may be somewhat limited by the immunosuppressive nature of “cold” tumors, and can be optimized by the addition of immunotherapies, such as anti-PD-1, to avoid tumor regrowth and better enable complete tumor elimination. This also suggests that THT could potentially rescue patients who fail PD-1 checkpoint inhibition by enhancing immune infiltration and activation.

## Conclusion

In summary, we have demonstrated that i.t. administration of GNRs, activated by a single exposure to NIR light, induces mild THT that preferentially triggers cancer cell death and activates ICD in the TME. . This subsequently stimulates a robust, tumor-specific adaptive (CD-8+ T cell) immune response. The addition of standard local (i.t.) immunotherapy such as i.t. IL-2, or systemic immunotherapy such as an anti-PD-1 inhibitor, further enhanced this immune response by shifting the TME towards a more pro-inflammatory anti-tumor environment, thereby promoting prolonged suppression of tumor growth. Our findings provide compelling proof of concept that patients failing current immunotherapies could potentially benefit from the inclusion of THT, converting their tumors from "cold" (immunologically unresponsive) to "hot" (responsive) tumors thereby improving the effectiveness of existing therapies.

## Limitations

One limitation of our study is the exclusive use of female mice, particularly in the B16-F10 melanoma model. While the use of female mice is justified in the 4T1 breast cancer model due to the low incidence of breast cancer in males, the absence of male mice in the B16-F10 experiments may limit the generalizability of our findings. Future studies should include both sexes to better understand sex-based differences in tumor biology and treatment responses.

The i.t. hyperthermia strategy employed in our study is currently best suited for superficial or easily accessible tumors due to the limited tissue penetration of NIR light (approximately 2 – 3 cm) and the challenges of injecting nanoparticles into deep tumors. However, advancements in nanoparticle engineering, such as functionalization with tumor-specific ligands or optimization for systemic delivery, could address these limitations. Multifunctional nanoparticles, combining imaging and therapeutic capabilities, as described by Li and Kataoka (67), may improve tumor specificity, real-time monitoring, and expand the method's applicability to deep or disseminated tumors (67). Future research should also explore alternative light delivery techniques, such as interstitial lasers, to enhance GNR activation in deep tissues.

## Data Availability

The raw data supporting the conclusions of this article will be made available by the authors, without undue reservation.
