# Supplementary material for: Targeted intra-tumoral hyperthermia using uniquely biocompatible gold nanorods induces strong immunogenic cell death in two immunogenically ‘cold’ tumor models
Source: Front Immunol. 2025 Jan 13;15:1512543. doi: 10.3389/fimmu.2024.1512543 (PMC11769938; doi:10.3389/fimmu.2024.1512543)
Supplement: Supplementary file 1 [file DataSheet1.docx]

# Supplemental Figures


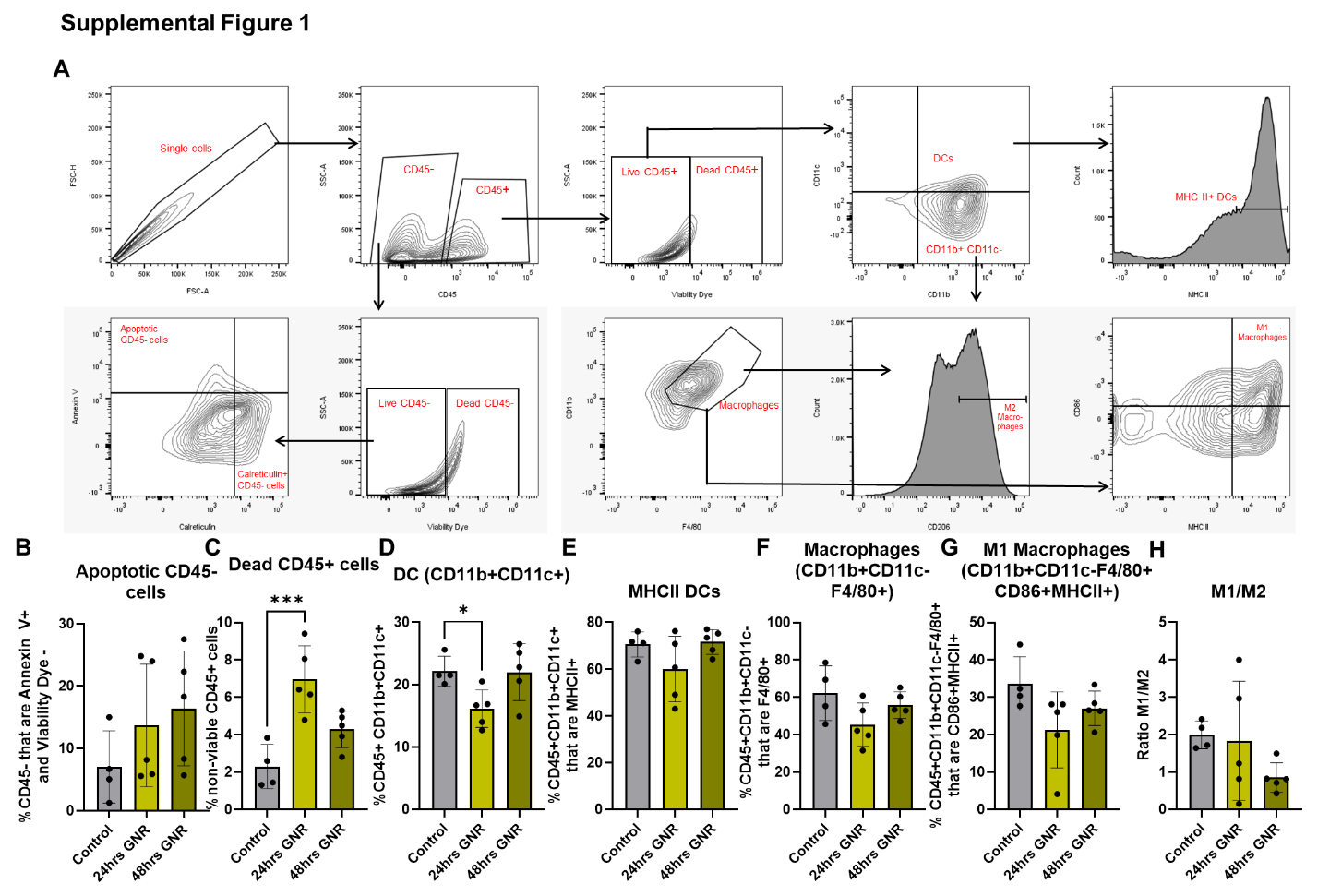


#
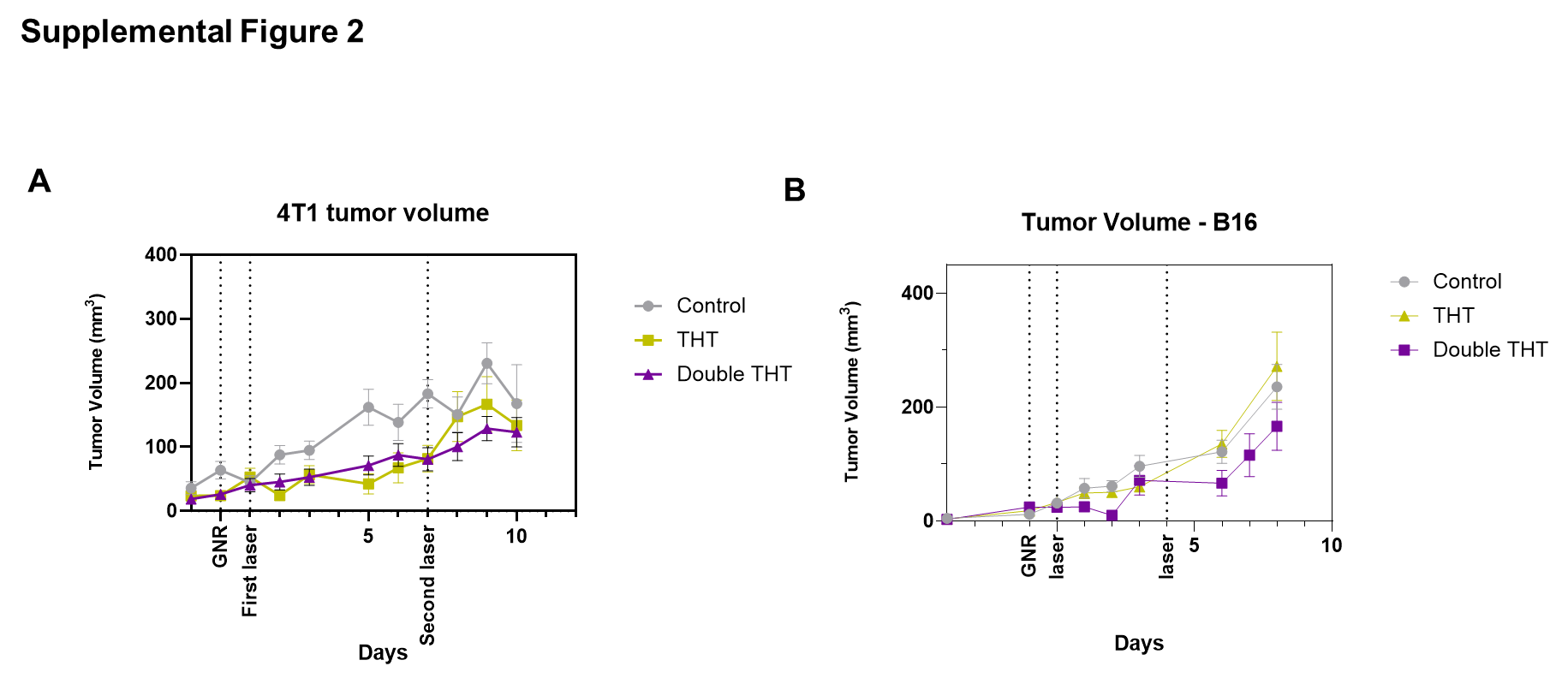


#
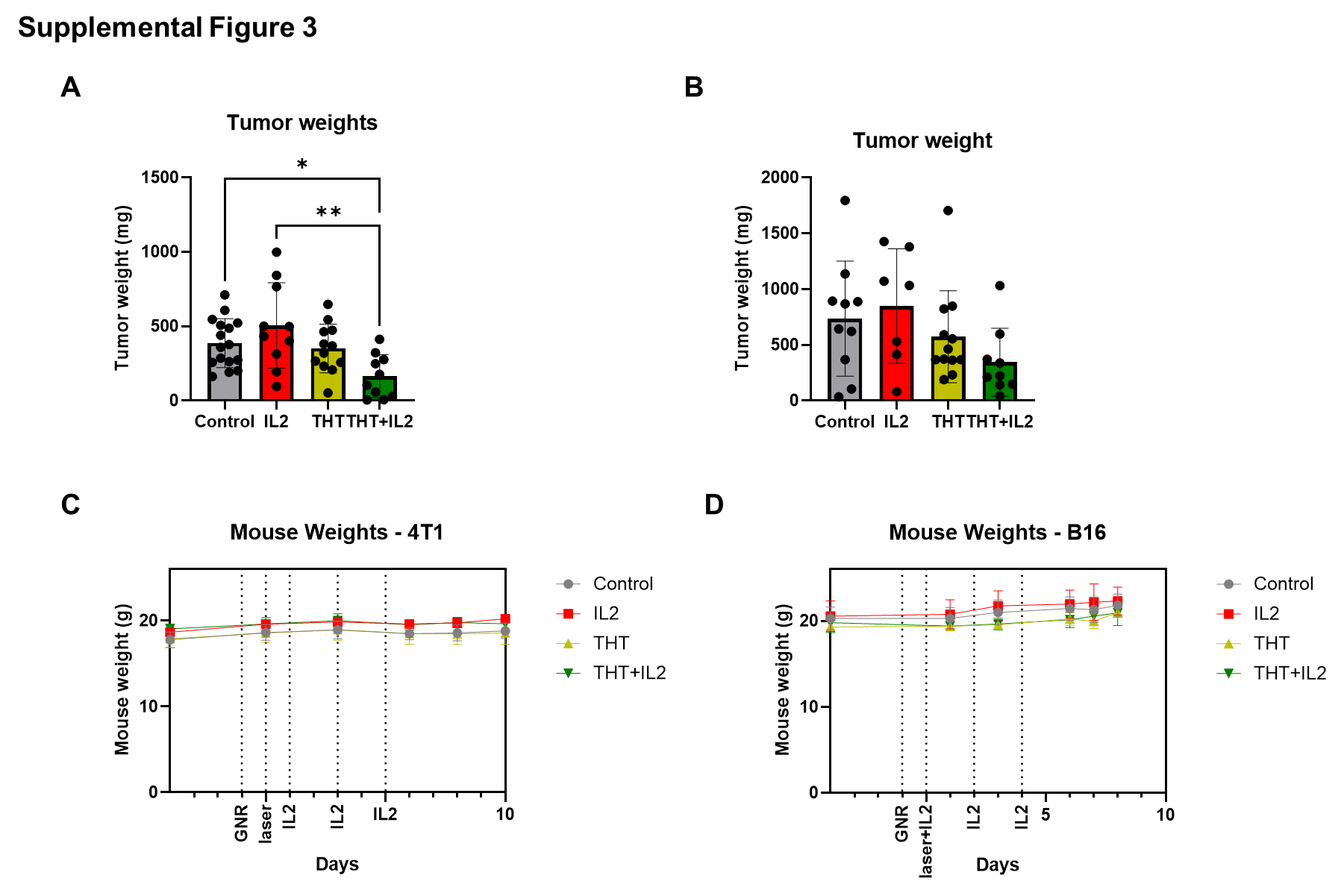


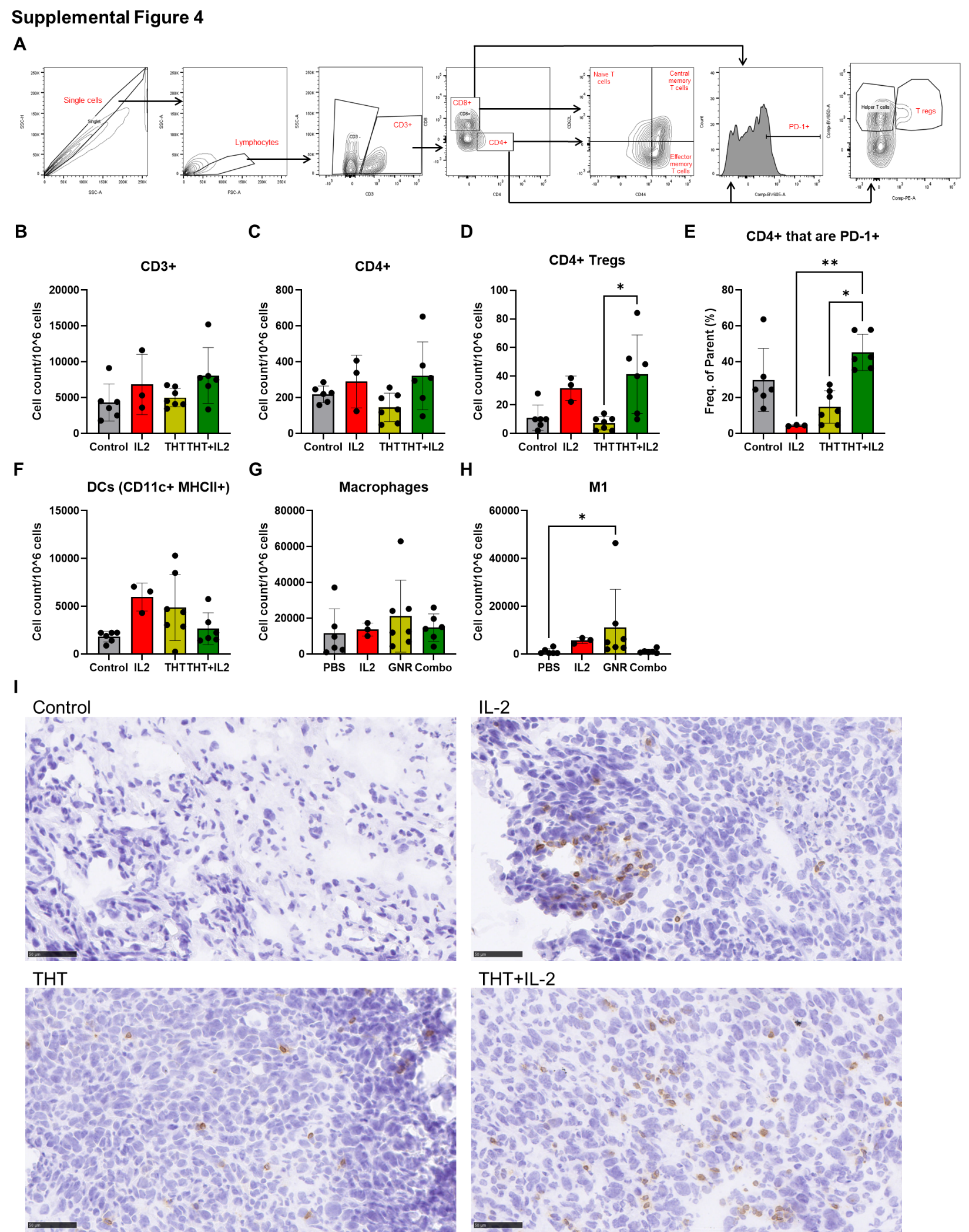


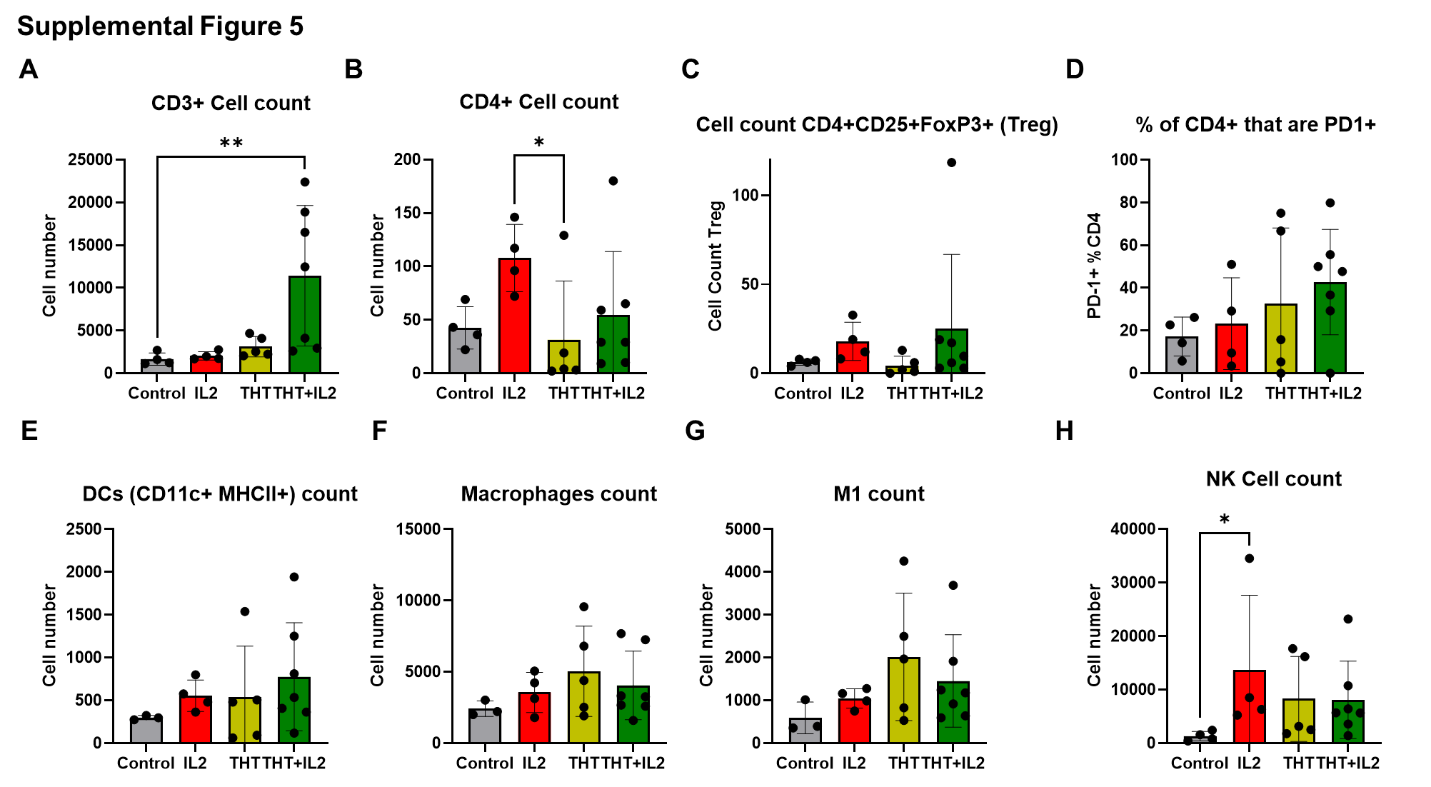


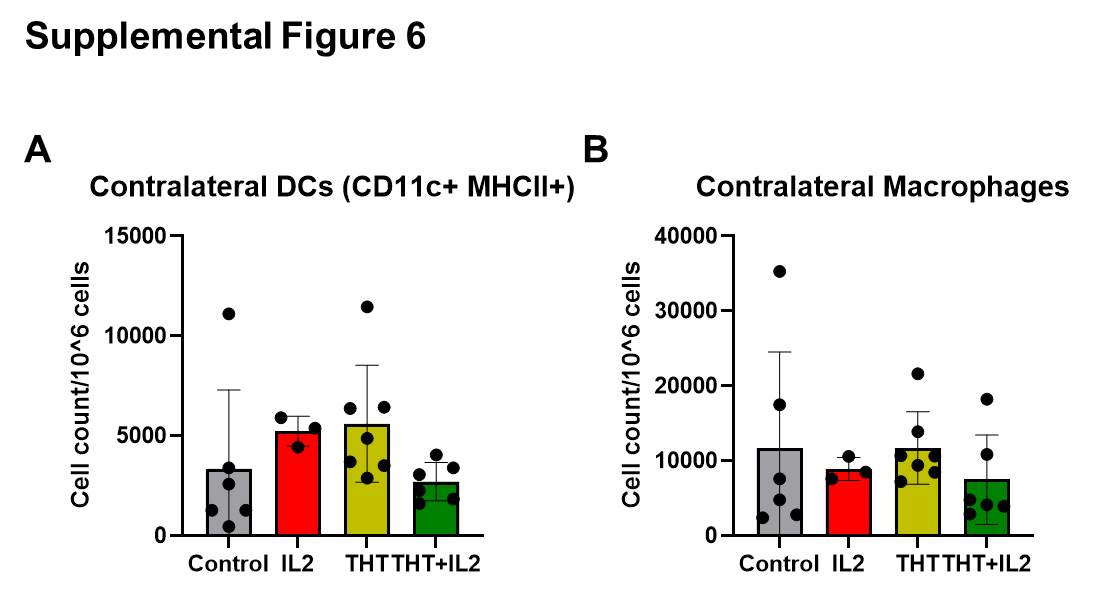


**Supplemental Figure Legends**

**Supplemental Figure 1. GNR-Mediated THT Induces Cell Death and Immune Activation in 4T1 Tumors Within 48 Hours Post-Treatment**

A. Gating strategy for myeloid and cell death panel. B. Analysis of apoptotic CD45- cells (gated on CD45-/viability dye- and annexin V +) in 4T1 tumors 24 and 48 hours post-THT (n=5). C. Dead CD45+ cells (gated on CD45+/viability dye+) in 4T1 tumors 24 and 48 hours post-THT (n=5). D. Dendritic cells (DCs, gated on viability dye-/CD45+/CD11b+/CD11c+) in 4T1 tumors 24 and 48 hours post-THT (n=5). E. Percentage of DCs that express MHCII (gated on viability dye-/CD45+/CD11b+/CD11c+/MHCII+) in 4T1 tumors 24 and 48 hours post-THT (n=5). F. Macrophages (gated on viability dye-/CD45+/CD11b+/CD11c-/F480+) in 4T1 tumors 24 and 48 hours post-THT (n=5). G. M1 Macrophages (gated on viability dye-/CD45+/CD11b+/CD11c-/F480+/CD86+/MHCII+) in 4T1 tumors 24 and 48 hours post-THT (n=5). H. M1/M2 ratio, n=5. Error bars represent standard deviation. ***p<0.001

**Supplemental Figure 2. Evaluation of Tumor Regrowth Following Single vs. Double THT Regimen in 4T1 and B16-F10 Models**

Effect of a double THT regimen on tumor volume in 4T1 (A) and B16-F10 (B) models. Mice were treated with either a single THT (initial NIR activation) or a double THT regimen, where a second NIR activation was applied 4 days after the first treatment for 4T1 tumors, and 7 days after for B16-F10 tumors (n=16 control, 15 single THT, 7 double THT). Error bars represent SEM.

**Supplemental Figure 3. Final Tumor and Mouse Weights Following THT and IL-2 Treatment in 4T1 and B16-F10 Models**

(A) Final tumor weights for the 4T1 model across the different treatment groups (n=16 control, 10 IL2, 13 THT, 11 THT+IL-2). (B) Final tumor weights for the B16-F10 model across the different treatment groups (n=9 control, 7 IL2, 10 THT, 10 THT+IL2). (C-D) Mouse body weights throughout the course of the experiments, indicating no significant changes in body weight between groups. Data are presented as mean ± SEM. *p < 0.05, **p < 0.01.

**Supplemental Figure 4. Flow cytometry analysis of immune cell infiltration and phenotype in 4T1 tumors treated with THT and i.t. IL-2**

Flow cytometry analysis of immune cell infiltration and phenotype in 4T1 tumors treated with THT and i.t. IL-2 (n= 6 Control, 3 IL2, 7 THT, 6 THT+IL2). (A) Gating scheme used for immune cell identification. (B) CD3+ T cell, (C) CD3+CD4+ T cell, and (D) Treg (CD3+CD4+FoxP3+CD25+) populations. (E) PD-1 expression on CD3+CD4+ T cells. (F-G) Dendritic cell (DC, CD45+/CD11b+/CD11c+) and macrophage (CD45+/CD11b+/CD11c-/F480+) populations, with (H) M1 macrophage levels (CD45+/CD11b+/CD11c-/F480+/CD86+/MHCII+). (I) Representative 40x images of immunohistochemistry staining of extracted tumor samples from control, i.t. IL-2, THT, and THT + i.t. IL-2 treated 4T1 tumors stained with hematoxylin and anti-CD3. Brown staining represents CD3+ cells. Scale bar represents 50 µm. *p < 0.05, **p < 0.01.

**Supplemental Figure 5. Flow Cytometry Analysis of Immune Cell Infiltration and Phenotype in B16-F10 Tumors Following THT and i.t. IL-2 Treatment**

Flow cytometry analysis of immune cell infiltration and phenotype in B16-F10 tumors treated with THT and i.t. IL-2 (n= 4 Control, 4 IL2, 5 THT, 7 THT+IL2). (A) CD3+ T cell, (B) CD4+ T cell, and (C) Treg (CD3+CD4+FoxP3+CD25+) populations. (D) PD-1 expression on CD3+CD4+ T cells. (E) Dendritic cell (DC, CD45+/CD11b+/CD11c+), (F) macrophage (CD45+/CD11b+/CD11c-/F480+) populations, and (G) M1 macrophage levels (CD45+/CD11b+/CD11c-/F480+/CD86+/MHCII+) with (H) NK cell (CD3-NK1.1+) levels. *p < 0.05, **p < 0.01.

**Supplemental Figure 6. Immune Cell Populations in Contralateral 4T1 Tumors Following GNR-Induced THT and IL-2 Treatment**

Panel (A) shows the quantification of dendritic cells (DCs, gated on CD11c+MHCII+) in contralateral 4T1 tumors across the different treatment groups (n=6 Control, 3 IL2, 6 THT, 6 THT+IL2). Panel (B) illustrates the quantification of macrophages (gated on CD45+/CD11b+F480+) in contralateral 4T1 tumors (n=6 Control, 3 IL2, 6 THT, 6 THT+IL2).
